# Co‐Ligand‐Induced Electronic Modulation Controls Substrate Adsorption and Selectivity in Biomass Electrooxidation

**DOI:** 10.1002/advs.77532

**Published:** 2026-09-02

**Authors:** Chen Gao, Bin Han, Shuai Chen, Shuai Yan, Ralf Weisbarth, Jorg Daniels, Nikolay Kornienko

**Affiliations:** ^1^ Institute of Inorganic Chemistry University of Bonn Bonn Germany; ^2^ University of Strasbourg CNRS ISIS UMR 7006 Strasbourg France

**Keywords:** attenuated total reflection, adsorption, catalysis, selectivity, reaction intermediate, faraday efficiency, electrocatalyst, chemical engineering, infrared spectroscopy, chemistry

## Abstract

The electrooxidation of 5‐hydroxymethylfurfural (HMF) to 2,5‐furandicarboxylic acid (FDCA) is often limited by competitive adsorption between HMF and surface‐bound hydroxyl species (OH_ads_), which governs both activity and selectivity, yet independent control over these interfacial adsorption processes remains a fundamental challenge. Here, we report a molecular design strategy that introduces distinct anionic co‐ligands into Cu‐based catalysts to modulate the electronic density of the metal center and, consequently, its interaction with both OH_ads_ and HMF. This approach enables differential tuning of competing adsorption processes on the same active sites, effectively reshaping their intrinsic coupling under fixed reaction conditions. As a result, the optimized catalyst achieves catalyst‐controlled pathway selectivity together with efficient FDCA production, delivering a Faradaic efficiency of 99%, a selectivity of 98%, and a high partial current density of 351 mA cm^−2^ at 1.6 V vs. RHE. In situ attenuated total reflectance (ATR) infrared spectroscopy reveals distinct adsorption behaviors and reaction intermediates, providing molecular‐level insight into the origin of selectivity. This work establishes co‐ligand engineering as an effective strategy for precise interfacial adsorption control in electrocatalysis, enabling catalyst‐controlled pathway selectivity beyond conventional regulation by pH and applied potential.

## Graphical abstract

A molecular design strategy is reported that introduces distinct anionic co‐ligands into Cu‐based catalysts to modulate the electronic density of the metal center and, consequently, its interaction with both OH_ads_ and HMF. The work establishes co‐ligand engineering as an effective strategy for precise interfacial adsorption control in electrocatalysis, enabling catalyst‐controlled pathway selectivity beyond conventional regulation by pH and applied potential.

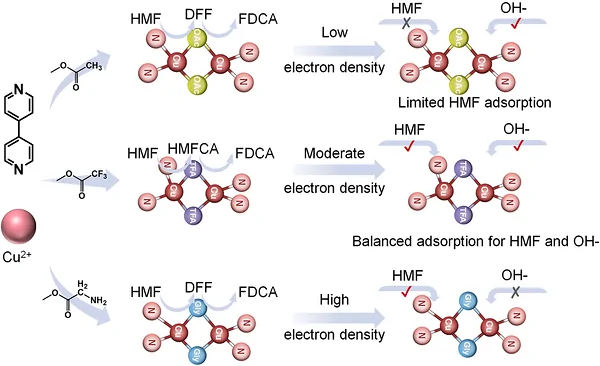

## 1 Introduction

The gradual depletion of fossil resources and the severe environmental issues arising from their excessive consumption have intensified the global pursuit of sustainable energy and chemical production [1, 2, 3]. In this context, the conversion of abundant and renewable biomass into value‐added chemicals and fuels offers a promising strategy to reduce reliance on fossil resources and mitigate greenhouse gas emissions [4, 5, 6, 7, 8, 9, 10, 11]. Among biomass‐derived platform molecules, 5‐hydroxymethylfurfural (HMF), featuring aldehyde and hydroxyl functionalities integrated within a furan ring, has emerged as a particularly attractive intermediate owing to its versatile chemical reactivity [12, 13, 14]. HMF is produced from the dehydration of hexoses (mainly fructose) or via acid‐catalyzed hydrolysis/dehydration of cellulose [15, 16, 17]. The oxidation of HMF to 2,5‐furandicarboxylic acid (FDCA) is of particular interest, as FDCA serves as the key monomer for polyethylene furanoate (PEF), a renewable, recyclable, and non‐toxic polyester with properties comparable to those of polyethylene terephthalate (PET) [18]. Despite this promise, conventional FDCA production relies predominantly on thermochemical processes operated under harsh conditions, including elevated temperatures and pressures [19]. Consequently, the electrochemical oxidation of HMF to FDCA under ambient conditions has attracted increasing attention as a sustainable and energy‐efficient alternative [20]. Recent advances in HMF oxidation reaction (HMFOR) electrocatalysis have been reported, particularly in catalyst design and interfacial engineering strategies [21, 22, 23].

**FIGURE 1:**
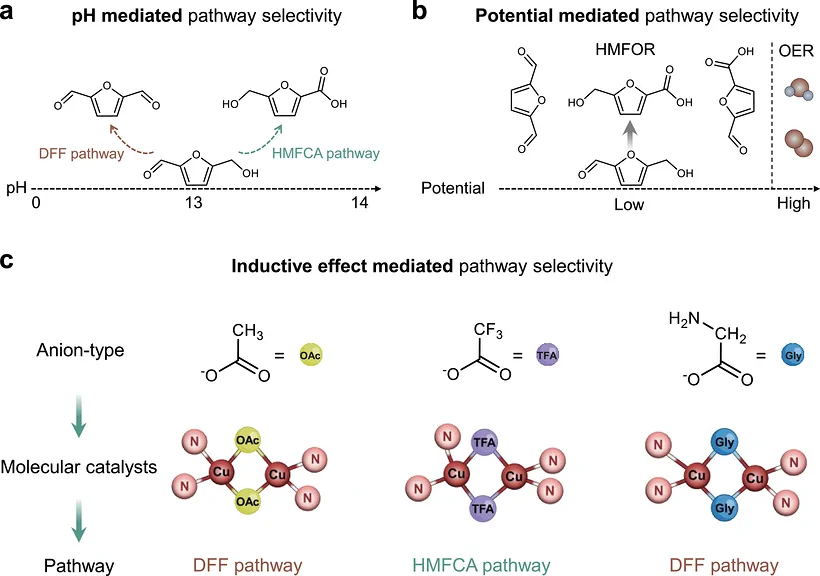
General concept of synthetic approach. (a) Schematic illustration of the Sabatier principle. (b) Conventional strategy in previous studies, where counter‐anions in the electrolyte are used to tune adsorption behavior. (c) The approach adopted in this work, where counter‐anions act as co‐ligands to directly regulate adsorption behavior.

The electrooxidation of HMF to FDCA is a six‐electron process proceeding via two competing pathways: oxidation of the hydroxymethyl group to DFF (2,5‐diformylfuran) or oxidation of the aldehyde group to HMFCA (5‐hydroxymethyl‐2‐furancarboxylic acid). Pathway selection is commonly dictated by electrolyte pH and applied potential. Strongly alkaline conditions favor the HMFCA route, while milder alkalinity promotes DFF formation, reflecting differences in functional group reactivity (Figure 1a,b) [24, 25]. Simultaneously, the mismatch between HMF and OH adsorption promotes the competing oxygen evolution reaction (OER), thereby undermining reaction selectivity. As OH_ads_ participates in each elementary step, FDCA production requires a precise balance: insufficient OH_ads_ limits oxidation, whereas excessive OH_ads_ suppresses HMF adsorption and promotes OER.

Significant efforts have been devoted to the competitive adsorption between HMF and surface‐bound OH_ads_ at active sites, including increasing surface area [26, 27], reducing particle size [28, 29], and introducing secondary active sites via doping [27, 30] or heterostructure construction [31, 32]. While these strategies can improve overall activity by tuning the global balance between HMF and OH_ads_, they generally treat HMF as a single adsorbate and overlook its multifunctional nature. In practice, the furan ring, aldehyde group, and hydroxymethyl group can interact with catalytic surfaces through distinct adsorption configurations, yet their respective competition with OH_ads_ remains poorly resolved.

To address this limitation, it is essential to develop catalyst systems capable of differentiating the adsorption of individual functional groups while independently balancing their interaction with OH_ads_. A co‐ligand engineering strategy was employed to construct three structurally analogous Cu‐based molecular catalysts, Cu(OAc)_2_BPY, Cu(TFA)_2_BPY, and Cu(Gly)_2_BPY (BPY: 4,4’‐ bipyridine, OAc: ─CH_3_COO, TFA: ─CF_3_COO, and Gly: ─NH_2_CH_2_COO), in which the coordination structure is preserved while the electron density of the Cu center is systematically tuned (Figure 1c). This electronic modulation regulates the competitive adsorption between OH_ads_ and substrates, while differentiating the adsorption of the hydroxymethyl and aldehyde groups of HMF on the same active site. Through in situ electrochemical impedance spectroscopy (EIS), substrate‐blocking experiments, single‐functional‐group probes, and in situ attenuated total reflectance (ATR) infrared spectroscopy (in situ ATR‐IR), we demonstrate that co‐ligand‐induced electronic effects break the conventional non‐selective adsorption balance and enable pathway‐selective HMFOR on the same active site under identical conditions. The optimized catalyst delivers a Faradaic efficiency of 99%, a selectivity of 98%, and a partial current density of 351 mA cm^−2^ at 1.6 V vs. RHE. The primary objective of this study is to clarify how the electronic density of the central metal governs substrate adsorption and thereby dictates the electrocatalytic oxidation pathway of HMF, providing fundamental design principles for achieving selective oxidation of HMF to FDCA.

## 2 Results

### 2.1 Tunable Cu Electronic Densities by Counter‐Anion Coordination

**FIGURE 2:**
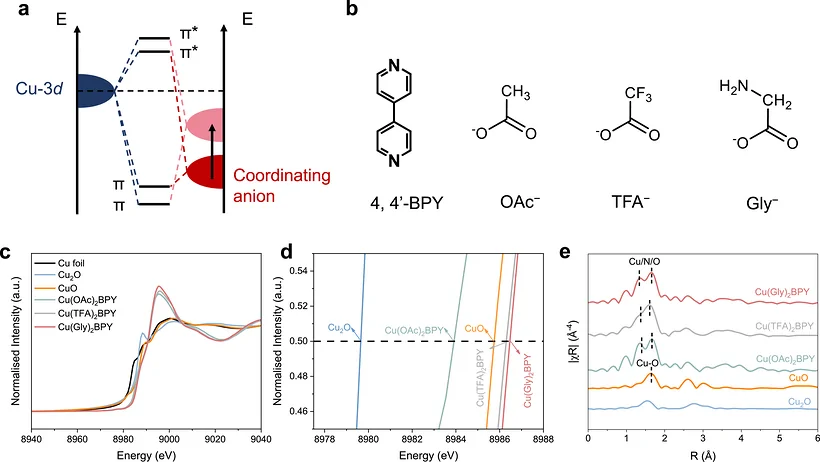
Different counter anions tune Cu electronic densities. (a) Schematic diagram of anion‐dependent modulation of Cu orbital energy levels. (b) Chemical structure of ligand molecules modelled. (c) Cu k‐edge normalized XANES and (d) Cu k‐edge EXAFS spectra in R space of Cu foil and CuO, respectively. (e) Cu k‐edge EXAFS spectra of the simulated R space.

Inspired by coordination chemistry, we modulate the electronic properties of the metal center by tuning the donor–acceptor characteristics of coordinated anions while preserving the primary ligand framework [33, 34, 35, 36]. The electron‐transfer propensity of the metal center is governed by the energy of its meta‐centered HOMO, which can be systematically tuned by the electronic nature of the coordinated co‐ligands (Figure 2a) [36]. Based on this strategy, three Cu‐based molecular catalysts with different co‐ligands, Cu(OAc)_2_BPY, Cu(TFA)_2_BPY, and Cu(Gly)_2_BPY, were synthesized via a self‐assembly approach using BPY and the corresponding organic salts (Figure 2b). Single‐crystal X‐ray diffraction analysis confirms the similar structures of Cu(TFA)_2_BPY and Cu(OAc)_2_BPY (Figure S1 and Table S1), The purities of the synthesized molecular catalysts were further confirmed by the X‐ray diffraction (XRD) patterns (Figure S2a–c). The scanning electron microscopy (SEM) and transmission electron microscopy (TEM) images clearly showed that the morphologies of Cu(OAc)_2_BPY, Cu(TFA)_2_BPY, and Cu(Gly)_2_BPY were rod‐like with progressively increasing rod size (Figures S3–S6). The X‐ray photoelectron spectroscopy (XPS) was further conducted to probe the electronic states of the Cu centers and the influence of different co‐ligands on the electronic structure of the catalysts (Figures S7–S10). The Fourier transform infrared spectra (FT‐IR) (Figures S11 and S11c), the characteristic peaks of ligands can be observed on these three molecular catalysts. Specifically, all three catalysts exhibit a characteristic ─COO^−^ symmetric stretching band at approximately 1417 cm^−1^ [37]. In addition, the band observed at ∼1375 cm^−1^ for Cu(OAc)_2_BPY can be assigned to the bending vibration of ─CH_3_ [38], while the band near ∼1100 cm^−1^ for Cu(TFA)_2_BPY corresponds to the bending vibration of ─CF_3_ [39, 40], and the feature around ∼900 cm^−1^ for Cu(Gly)_2_BPY is attributed to the bending vibration of ─NH_2_ [41]. These features indicate that the three anions are directly involved in coordination with the metal center. Overall, the three catalysts possess similar coordination structures, differing only in the nature of the anions bound to the copper center.

The electronic structures of the three catalysts were investigated using synchrotron‐based X‐ray absorption spectroscopy (XAS). The absorption edge position of Cu(OAc)_2_BPY in the Cu K‐edge X‐ray absorption near‐edge structure (XANES) spectrum is located close to that of CuO, indicating an oxidation state of +2 for the copper center (Figure 2c) [34]. In addition, a distinct K‐edge pre‐edge feature is observed for Cu(OAc)_2_BPY, which is characteristic of Cu^2+^ species [34]. The position of half height (0.5) of the normalized XANES spectrum is further utilized to act as the absorption edge to avoid ambiguous identification of the position of the absorption edge [42]. Notably, a gradual decrease in the Cu oxidation state from Gly and TFA to OAc is evidenced by the systematic shift of the Cu K‐edge to lower energies (Figure 2d). The ─CH_3_ group in OAc acts as an electron‐donating substituent, thereby stabilizing the copper center in a lower oxidation state. In contrast, the strongly electron‐withdrawing ─CF_3_ group in TFA promotes a higher effective oxidation state of copper. For Gly, its side group can participate in *π–π* electronic conjugation between the metal center and the counter anions, further enhancing electron delocalization and leading to the highest effective oxidation state among the three catalysts. These ligand‐induced electronic effects are fully consistent with the observed XANES results. Fourier transform extended X‐ray absorption fine structure (FT‐EXAFS) analysis [43], together with single‐crystal X‐ray diffraction data, reveals that the Cu center in Cu(TFA)_2_BPY adopts a five‐coordinate geometry, coordinated by two N atoms and three O atoms (Figure 2e and Table S1). In contrast, Cu(OAc)_2_BPY and Cu(Gly)_2_BPY exhibit six‐coordinate Cu centers, each coordinated by two N atoms and four O atoms. The FT‐EXAFS spectra of all three catalysts display two dominant peaks at approximately 1.35 and 1.56 Å, which can be assigned to the first‐shell Cu─N and Cu─O scattering paths [44, 45].

Importantly, the catalytic selectivity cannot be directly correlated with the coordination geometry As shown in Figures S7 and S8, the Cu 2p_3/2_ peak gradually shifted toward higher binding energy from Cu(OAc)_2_BPY to Cu(TFA)_2_BPY and Cu(Gly)_2_BPY, indicating a progressive decrease in the electron density of Cu centers. Consistently, the Cu LMM Auger peak also shifted from 576.3 to 576.9 eV, further confirming the electron‐withdrawing effect of the co‐ligands and the generation of more electron‐deficient Cu sites. As shown in Figures S9 and S10, the N 1s and O 1s spectra exhibit similar peak profiles for all samples, while slight positive shifts are observed for Cu(TFA)_2_BPY and Cu(Gly)_2_BPY relative to Cu(OAc)_2_BPY. This trend suggests a reduced electron density in the coordination environment, consistent with the Cu 2p and Cu LMM results. Overall, these spectroscopic results consistently demonstrate that the electronic structure of the Cu centers can be systematically tuned by the nature of the co‐ligands.

### 2.2 Study on Interfacial Adsorption Kinetics

After confirming the successful synthesis of the target catalysts, we first evaluated their electrocatalytic performance, as establishing catalytic activity is a prerequisite to subsequent mechanistic studies of adsorption behavior and reaction pathways. Unless otherwise specified, all potentials are referenced to the reversible hydrogen electrode (RHE). In the presence of HMF, all three catalysts exhibit more positive oxidation currents than the OER (Figure S12a,b), indicating kinetically favorable HMFOR. The catalytic activity shows a volcano‐type dependence on Cu‐center electron density. Cu(TFA)_2_BPY, with intermediate electron density, delivers the lowest onset potential of 1.296 V, outperforming Cu(Gly)_2_BPY (1.375 V) and Cu(OAc)_2_BPY (1.489 V). Tafel analysis further supports the superior kinetics of Cu(TFA)_2_BPY, which exhibits the smallest slope 126 mV dec^−1^, compared with Cu(Gly)_2_BPY (127 mV dec^−1^) and Cu(OAc)_2_BPY (145 mV dec^−1^, Figure S13), indicating superior HMFOR kinetics. Double‐layer capacitance measurements reveal the electrochemically active surface area (ECSA) values among the three catalysts (Figure S14a–f), consistent with their similar structures differing only in anionic co‐ligands. Notably, the HMFOR activity of Cu(TFA)_2_BPY is comparable to that of state‐of‐the‐art Cu‐based catalysts and even surpasses some previously reported noble‐metal‐doped catalysts (Table S2).

To probe product distribution, nuclear magnetic resonance (NMR) spectroscopy was employed for the Cu(TFA)_2_BPY system. According to stoichiometric calculations, complete conversion of 10 mm HMF in 10 mL KOH electrolyte to FDCA theoretically requires a total charge of approximately 57.8 C. As charge accumulation increased, HMF was progressively converted to FDCA, with only trace HMFCA and FFCA intermediates detected (Figure S15a). Notably, the Faradaic efficiency (FE) toward FDCA reaches 99%, while the corresponding partial current density (PCD) 50 mA cm^−2^, indicating nearly quantitative conversion of HMF into the high‐value product FDCA, both higher than those of Cu(Gly)_2_BPY (FE = 91%, PCD = 25 mA cm^−2^) and Cu(OAc)_2_BPY (FE = 56%, PCD = 4 mA cm^−2^), respectively. (Figure S15b). Furthermore, the Cu(TFA)_2_BPY catalyst exhibits negligible performance decay over six consecutive catalytic cycles (Figure S16). Moreover, the solution color gradually faded from yellow to colorless during HMF oxidation, as captured in photographs at different stages (Figure S17a–c). Post‐reaction SEM, FT‐IR, XRD, XAS analyses, and Inductively Coupled Plasma‐Optical Emission Spectroscopy (Figures S18–S21 and Table S3) suggest that the structural integrity of Cu(TFA)_2_BPY is well preserved after HMFOR, indicating its excellent catalytic stability. These results further highlight the crucial role of an optimized anionic co‐ligand in enabling highly selective conversion. Having established the superior catalytic performance of the catalysts, we next sought to identify the interfacial factors governing their enhanced activity. As shown in Figure S22, upon addition of 10 mm KSCN, the HMF oxidation peak was significantly suppressed and nearly disappeared, indicating a strong poisoning effect on the catalytic activity and suggesting that Cu‐based sites are responsible for HMFOR.

**FIGURE 3:**
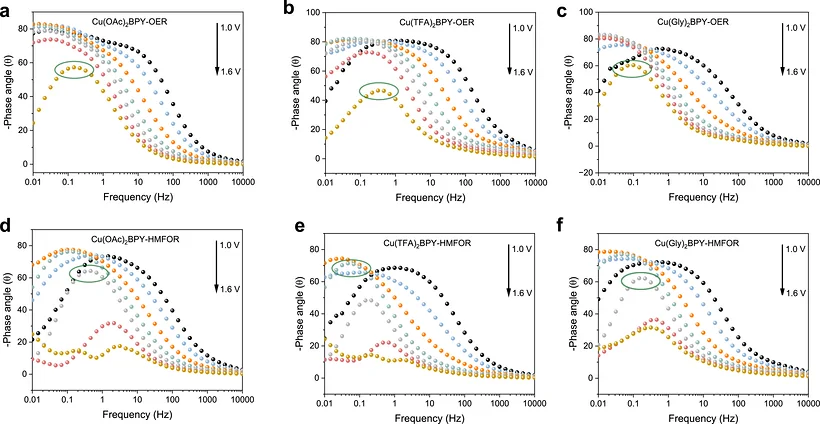
Study on interfacial adsorption kinetics. Bode plots of the Cu(OAc)_2_BPY, Cu(TFA)_2_BPY and Cu(Gly)_2_BPY catalysts in (a–c) 0.1 m KOH and (d–f) 0.1 m KOH + 10 mm HMF.

We first employed in situ EIS to evaluate the interfacial electrochemical behavior during HMFOR [46]. The corresponding Bode plots, which depict the evolution of the phase angle as a function of frequency, are presented in Figure 3a–f. The Bode plots can be divided into two characteristic regions: a high‐frequency region (> 2 Hz) and a low‐frequency region (< 2 Hz). According to previous reports, the response in the high‐frequency region is mainly associated with electronic conduction within the catalyst layer and/or at the catalyst–substrate interface, whereas the low‐frequency region is generally attributed to interfacial charge‐transfer processes occurring at the catalyst‐electrolyte interface. As shown in Figure 3a–c, for Cu(OAc)_2_BPY, Cu(TFA)_2_BPY, and Cu(Gly)_2_BPY, the inflection point (marked by green circles), defined as the frequency region exhibiting a pronounced change in curvature in the low‐frequency domain, emerges at approximately 1.60 V upon increasing the applied potential. This feature is indicative of the onset of the OER. Notably, Cu(TFA)_2_BPY exhibits a lower phase angle in the high‐frequency region compared to Cu(OAc)_2_BPY and Cu(Gly)_2_BPY, suggesting more favorable charge transport characteristics and faster interfacial kinetics. Upon the introduction of HMF into the electrolyte (Figure 3d–f), the inflection points of Cu(OAc)_2_BPY and Cu(Gly)_2_BPY shift to around 1.40 V, whereas that of Cu(TFA)_2_BPY appears at a lower potential of 1.30 V, indicating a reduced onset potential for HMF oxidation on Cu(TFA)_2_BPY. Furthermore, within the potential window relevant to HMF oxidation (1.30–1.60 V), Cu(TFA)_2_BPY consistently displays a lower phase angle in the low‐frequency region than Cu(OAc)_2_BPY and Cu(Gly)_2_BPY, reflecting accelerated interfacial charge transfer and thus faster HMFOR kinetics. The corresponding equivalent circuit models and Nyquist plots are provided in Figures S23 and S24.

### 2.3 Adsorption of Reactants and Intermediates

**FIGURE 4:**
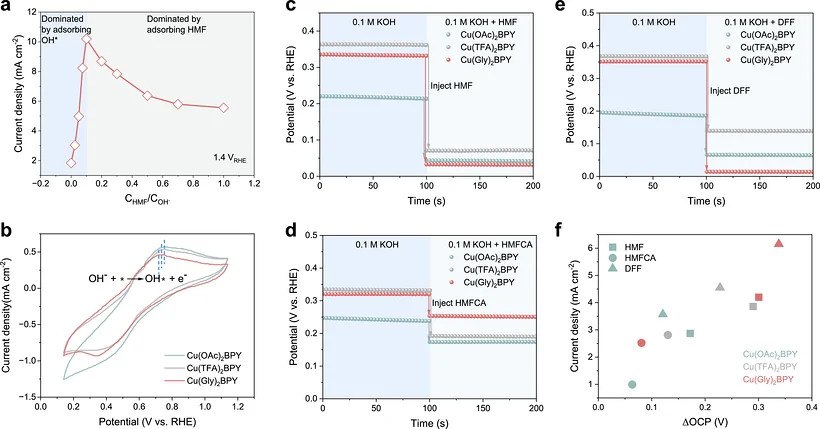
Investigation of the adsorption behavior for substrates. (a) Relationship between the current density and C_HMF_/C_OH_^−^ at 1.4 V. (b) CV curves of Cu(OAc)_2_BPY, Cu(TFA)_2_BPY and Cu(Gly)_2_BPY in 1.0 m PBS (pH 7) aqueous solution. OCP changes of Cu(OAc)_2_BPY, Cu(TFA)_2_BPY and Cu(Gly)_2_BPY in 0.1 m KOH aqueous solution after (c) HMF, (d) DFF and (e) HMFCA injected. (f) Conclusion on adsorption capacities of Cu(OAc)_2_BPY, Cu(TFA)_2_BPY and Cu(Gly)_2_BPY toward HMF, DFF and HMFCA.

HMF oxidation can proceed via either a direct or an indirect oxidation pathway. To clarify the mechanism on the present catalyst, concentration‐dependent electrochemical measurements were performed [47, 48]. As shown in Figure 4a, the current density exhibits a volcano‐shaped dependence on the C_HMF_/C_OH_^−^ ratio, indicating competitive adsorption between adsorbed HMF^*^ and OH_ads_ species. At low C_HMF_/C_OH_^−^ ratios, increasing HMF enhances the current due to sufficient OH_ads_ availability. Whereas at high C_HMF_/C_OH_^−^ ratios, excessive HMF adsorption blocks OH_ads_ sites and suppresses activity. This behavior supports a direct oxidation pathway, in which HMF is oxidized via interaction with adjacent surface OH_ads_ species. Based on these results, a mechanistic scheme was proposed for Cu(TFA)_2_BPY (Figure S25), where HMF to FDCA conversion proceeds through stepwise oxidation involving co‐adsorption of intermediates and OH_ads_, which serves as the oxygen source for dehydrogenation of alcohol and aldehyde groups.

Therefore, elucidating the adsorption behavior of OH_ads_ is crucial for understanding the HMFOR kinetics. The adsorption activity of OH_ads_ was evaluated under a hydroxide‐deficient environment using CV measurements conducted in 1.0 M phosphate‐buffered saline (PBS, pH 7). As shown in Figure 4b, a distinct oxidation peak appears in the potential window of 0.8–1.1 V, which is consistent with the reported potential region for OH_ads_ formation [49, 50]. Accordingly, this feature can be assigned to the oxidation of OH^−^ to OH_ads_ (OH^−^→OH_ads_ + e^−^). Among the three catalysts, Cu(OAc)_2_BPY (0.849 V) exhibits the strongest OH_ads_ affinity, followed by Cu(TFA)_2_BPY (0.829) with moderate adsorption strength, whereas Cu(Gly)_2_BPY (0.819 V) shows the weakest OH_ads_ adsorption.

To further probe the adsorption behavior of HMF and intermediates, open‐circuit potential (OCP) measurements were performed. OCP shifts reflect changes in the inner Helmholtz layer and can provide insight into relative interfacial adsorption behavior and catalyst‐adsorbate interactions [1, 51, 52, 53, 54]. Upon successive addition of 10 mm HMF, DFF, and HMFCA, all catalysts exhibit evident potential shifts, confirming their adsorption within the inner Helmholtz layer. As shown in Figure 4c, HMF induces comparable OCP shifts for Cu(TFA)_2_BPY (0.291 V) and Cu(Gly)_2_BPY (0.299 V), both larger than that of Cu(OAc)_2_BPY (0.209 V), suggesting stronger HMF adsorption on the former two catalysts. For HMFCA (Figure 4e), Cu(TFA)_2_BPY (0.128 V) exhibits the largest OCP shift, followed by Cu(Gly)_2_BPY (0.078 V) and Cu(OAc)_2_BPY (0.064 V), indicating relatively stronger adsorption of HMFCA on Cu(TFA)_2_BPY. In contrast, DFF adsorption (Figure 4d) follows Cu(Gly)_2_BPY > Cu(TFA)_2_BPY > Cu(OAc)_2_BPY. Notably, DFF consistently induces larger OCP shifts than HMFCA across all catalysts. Collectively, these results indicate that co‐ligands modulate the electronic structure of the Cu center, leading to distinct adsorption strengths toward reactants and intermediates. An appropriate balance between substrate and OH_ads_ binding is therefore essential for efficient HMFOR. As summarized in Figure 4f, the integrated ΔOCP values provide a direct comparison of adsorption tendencies and correlate positively with catalytic current. This trend suggests that adsorption plays an important role in determining HMFOR performance. However, because ΔOCP mainly reflects interfacial adsorption rather than intrinsic surface reaction kinetics, the observed correlation should be interpreted as evidence of adsorption‐controlled behavior rather than direct causality between ΔOCP and catalytic activity.

To further probe the interaction between the catalyst and HMF, in situ ATR‐FTIR spectroscopy was performed (Figure S26). The characteristic adsorption band of HMF appeared at 1145 cm^−1^ on Cu(TFA)_2_BPY and shifted to 1130 cm^−1^ on Cu(Gly)_2_BPY. The red shift of the adsorption band indicates a stronger interaction between HMF and Cu(Gly)_2_BPY compared with Cu(TFA)_2_BPY. In contrast, no obvious HMF adsorption signal was detected on Cu(OAc)_2_BPY, suggesting that it exhibits the weakest HMF adsorption capability among the three catalysts. These results suggest that ligand modulation influences the adsorption behavior of HMF on the catalyst surface, which may contribute to the observed differences in catalytic performance.

### 2.4 Adsorption Modes of HMF on Catalyst Surfaces

**FIGURE 5:**
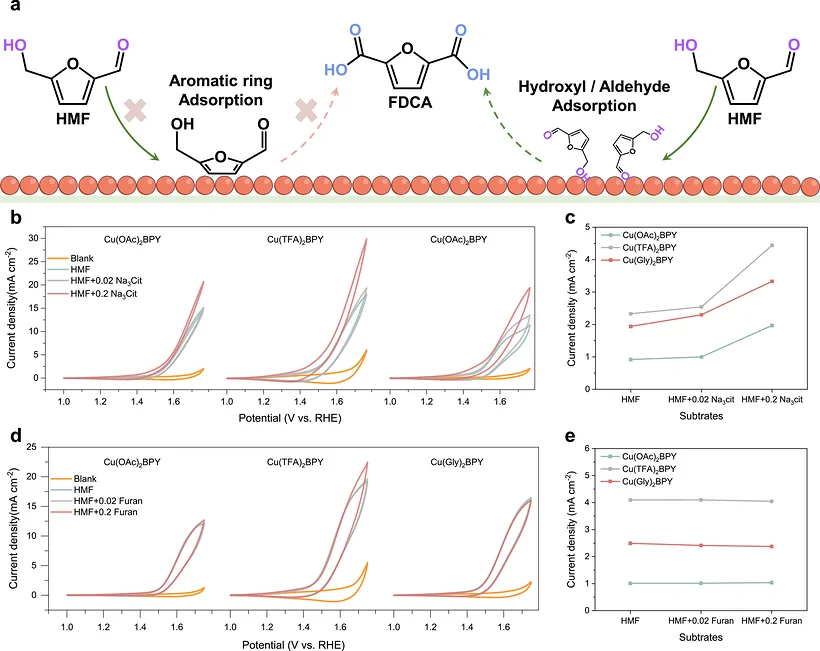
Exploring the adsorption models of HMF molecules. (a) Schematic illustration of the adsorption models of HMF molecules. (b, d) Cyclic curves of Cu(OAc)_2_BPY, Cu(TFA)_2_BPY, and Cu(Gly)_2_BPY with varying concentrations of citrate shown as their ratio of citrate or furan concentration and reactant. The blank recorded in 0.1 m KOH, and the HMFOR recorded in 20 mm HMF. (c, e) Current density of Cu(OAc)_2_BPY, Cu(TFA)_2_BPY and Cu(Gly)_2_BPY in the presence of different substrates at 1.5 V.

While OCP variations indicate the occurrence of interfacial adsorption, they do not distinguish the specific adsorption configuration or the dominant adsorbate species. The interaction of HMF with catalyst surfaces can proceed through distinct adsorption modes. HMF adsorption occurs via the furan ring or functional groups (─CHO/─CH_3_OH) [55], as shown in Figure 5a. To probe the adsorption configuration at the catalyst‐electrolyte interface, citrate (bearing oxygenated groups) and furan were employed as competitive probes for functional‐group and ring adsorption, respectively. If HMF adsorbs via its ─CHO/─CH_2_OH groups, the introduction of citrate is expected to perturb the current response; conversely, adsorption through the furan ring should be sensitive to the addition of furan. As shown in Figure 5b,c, adding citrate induces a concentration‐dependent increase in current density at 1.5 V, indicating competitive adsorption between citrate and HMF. In contrast, the introduction of furan causes negligible changes in both CV curves and current density (Figure 5d,e), regardless of its concentration. These results demonstrate that HMF predominantly adsorbs via its ─CHO/─CH_2_OH functional groups rather than the furan ring, consistent with previous reports [55, 56, 57].

### 2.5 Reaction Pathways from In Situ ATR‐IR

**FIGURE 6:**
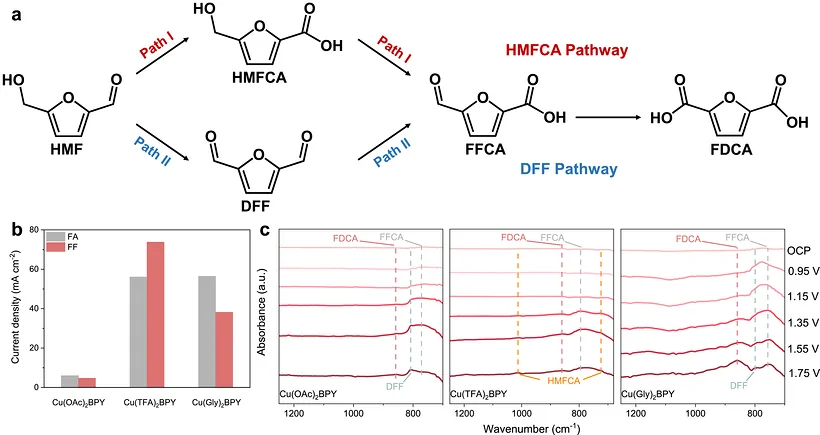
Investigations of HMFOR mechanism. (a) Two possible reaction pathways for HMF oxidation into FDCA. (b) Current density on three catalysts with FF and FA. (c) In situ ATR‐IR spectra of Cu(OAc)_2_BPY, Cu(TFA)_2_BPY, and Cu(Gly)_2_BPY.

In general, two possible reaction pathways have been proposed for the electrooxidation of HMF to FDCA (Figure 6a). To verify the oxidation pathways of HMF and further differentiate the adsorption behavior of its functional groups on different catalysts, electrochemical analyses were first conducted using furfural (FF) and furfuryl alcohol (FA) as model substrates. As shown in Figure 6b, Cu(OAc)_2_BPY exhibits poor activity toward both FF and FA, indicating limited catalytic capability for either functional group and, consequently, unfavorable HMF oxidation performance, consistent with the results in Figure S12a. In contrast, Cu(TFA)_2_BPY and Cu(Gly)_2_BPY show significantly enhanced oxidation activity, with Cu(Gly)_2_BPY favoring ─OH oxidation over ─CHO oxidation, whereas Cu(TFA)_2_BPY favors ─CHO oxidation over ─OH oxidation. To elucidate the HMF oxidation pathways, in situ ATR‐IR measurements were performed. Representative ATR‐IR spectra recorded in 10 mm HMF and 0.1 m KOH are shown in Figure S27 and Figure 6c. As shown in Figure S27, the O–H stretching bands (3100–3500 cm^−1^) display comparable intensities on Cu(OAc)_2_BPY and Cu(TFA)_2_BPY, both markedly stronger than on Cu(Gly)_2_BPY, indicating enhanced accumulation of OH_ads_ [58, 59], in agreement with the adsorption behavior in Figure 4b.

Furthermore, as shown in Figure 6c, the evolution of reaction intermediates during electrooxidation was monitored in situ over a potential range of 0.95–1.75 V. By comparison with reference spectra of standard compounds collected under identical conditions (Figure S28 and Table S4), the bands at 721 and 1010 cm^−1^ can be assigned to HMFCA. In particular, the gradual increase in the intensity of the 721 cm^−1^ band indicates the continuous formation and accumulation of HMFCA. The characteristic band at 860 cm^−1^ corresponds to FDCA, whose increasing intensity with electrooxidation time confirms the progressive generation of FDCA. The band at 757 cm^−1^ matches well with the reference spectrum of FFCA, and its intensity growth reflects the accumulation of FFCA. In addition, the absorption band at 796 cm^−1^ is attributed to DFF, with its increasing intensity indicating the gradual formation of DFF during electrooxidation. To further verify that the observed intermediate distribution is not an artifact of the measurement timing, time‐resolved in situ ATR‐FTIR measurements were performed on Cu(TFA)_2_BPY during HMFOR (Figure S29). The characteristic bands assigned to HMFCA, FFCA, and FDCA evolved progressively with electrolysis time, consistent with the sequential oxidation pathway proposed above.

Notably, only HMFCA‐related signals are observed on Cu(TFA)_2_BPY, while no DFF signal is detected, indicating that HMF oxidation predominantly proceeds via pathway I (aldehyde‐side oxidation). In contrast, Cu(OAc)_2_BPY and Cu(Gly)_2_BPY exhibit only DFF‐related signals without detectable HMFCA, suggesting a dominant pathway II (hydroxymethyl oxidation). These results are fully consistent with the model substrate experiments. Among the catalysts, Cu(OAc)_2_BPY shows the poorest performance due to overly strong OH_ads_ adsorption and insufficient HMF adsorption. Although Cu(Gly)_2_BPY enables selective DFF formation via enhanced hydroxymethyl adsorption, its limited OH_ads_ adsorption still constrains overall activity. In contrast, Cu(TFA)_2_BPY achieves an optimal balance between aldehyde adsorption and OH_ads_ interaction, leading to the highest activity and selectively directing the reaction toward the HMFCA pathway.

### 2.6 Electrochemical Performance and Substrate Scope of the Cu(TFA)_2_BPY Catalyst in a Flow Cell

**FIGURE 7:**
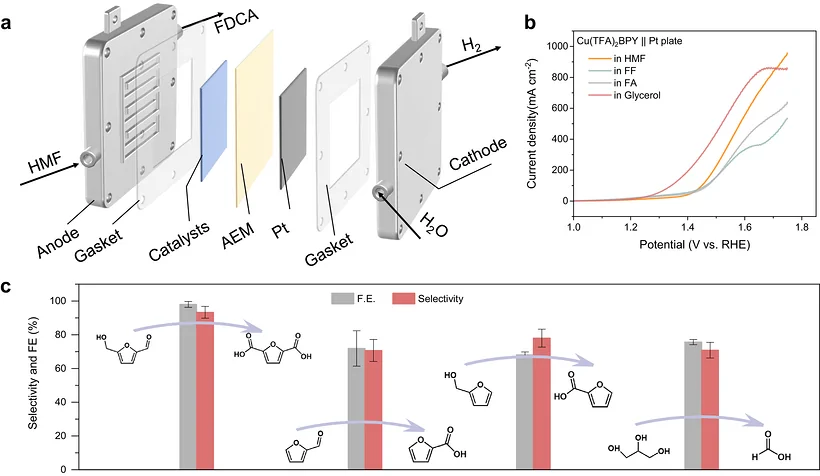
Scale‐up and substrate extension. (a) The visualized flow cell setup for HMFOR and HER. (b) LSV curves of Cu(TFA)_2_BPY measured with an expanded substrate scope at high substrate concentrations (100 mm HMF, FF, FA, and glycerol), using Pt as the cathode. (c) FE and selectivity of four substrates.

To evaluate the electrochemical performance of the Cu(TFA)_2_BPY catalyst under practical operating conditions and its substrate generality, a flow‐cell electrolysis configuration was employed, as illustrated in Figure 7a. In addition to HMF, several representative biomass‐derived alcohols and aldehydes were investigated, including furfural (FF), furfuryl alcohol (FA), and glycerol. The LSV curves recorded in the flow cell for different substrates are compared in Figure 7b and Figure S30, revealing distinct current responses and onset potentials depending on the substrate. In the absence of any organic substrate, the flow cell required a high input voltage of 1.8 V to reach a current density of 200 mA. By contrast, upon the introduction of HMF, FF, FA, and glycerol, the polarization curves shifted toward lower voltages by approximately 600, 400, 300, and 500 mV, respectively, at the same current density, indicating a substantial reduction in energy input and accelerated anodic oxidation kinetics. Notably, for the HMF system, a partial current density of 351 mA cm^−2^ was achieved at a cell voltage of 1.6 V, indicating the excellent high‐rate performance of the catalyst in the flow‐cell configuration (Figure S31). As shown in Figure S32, the FE remained above 80% during the first 6 h of continuous electrolysis, indicating good operational stability and product selectivity under flow‐cell conditions. A gradual decrease in FE was observed during prolonged operation. Beyond electrochemical activity, quantitative analysis of the oxidation products was performed. As shown in Figure 7c, Cu(TFA)_2_BPY exhibits high Faradaic efficiency and product selectivity for all investigated biomass‐derived substrates. Specifically, the oxidation of HMF to FDCA proceeds with a Faradaic efficiency of 95% and a selectivity of 93%. Both furfural and furfuryl alcohol are selectively converted to furoic acid, while glycerol oxidation predominantly yields formic acid, with Faradaic efficiencies exceeding 80% in all cases. These results collectively demonstrate the robustness and practical applicability of the Cu(TFA)_2_BPY catalyst toward diverse biomass‐derived substrates in a flow‐cell configuration.

## 3 Discussion

We demonstrate a ligand‐steered electronic modulation strategy to address pathway selectivity challenges under identical pH and applied potential. By employing a series of copper catalysts with tailored anionic co‐ligands, we successfully decouple the competitive adsorption of OH_ads_ and HMF at the Cu center. This structure‐electronics‐adsorption correlation, validated by in situ EIS, OCP, and in situ ATR‐IR, reveals that selectivity is governed by electronic‐density‐dictated functional group recognition rather than steric effects. Furthermore, competitive adsorption probes and model substrate studies further distinguish the adsorption modes on the catalyst surface. Our molecular platform achieves a near‐unity Faradaic efficiency (99%), selectivity (98%), and a high partial current density of 351 mA cm^−2^ at 1.6 V vs. RHE for HMFOR, offering a programmable blueprint for designing electrocatalysts with tunable reaction trajectories.

## Author Contributions

**Bin Han**: methodology, writing – review and editing, formal analysis, data curation. **Shuai Yan**: formal analysis, supervision. **Jorg Daniels**: methodology. **Chen Gao**: conceptualization, investigation, formal analysis. **Nikolay Kornienko**: conceptualization, funding acquisition, supervision. **Shuai Chen**: supervision. **Ralf Weisbarth**: methodology.

## Conflicts of Interest

The authors declare no conflicts of interest.

## Supporting information




**Supporting File**: advs77532‐sup‐0001‐SuppMat.pdf.

## Data Availability

The data that support the findings of this study are available from the corresponding author upon reasonable request.
