# Supplementary material for: Co‐Ligand‐Induced Electronic Modulation Controls Substrate Adsorption and Selectivity in Biomass Electrooxidation
Source: Adv Sci (Weinh). 2026 Sep 2:e77532. Online ahead of print. doi: 10.1002/advs.77532 (PMC13535540; doi:10.1002/advs.77532)
Supplement: Supplementary file 1 [file ADVS-9999-e77532-s001.pdf]

Supplementary information for

Co-Ligand-Induced Electronic Modulation Controls Substrate Adsorption and Selectivity in Biomass Electrooxidation

Chen Gao[1], Dr Bin Han[2], Dr. Shuai Chen[1], Shuai Yan[1], Dr. Ralf Weisbarth[1], Dr. Jorg Daniels[1], Prof. Dr. Nikolay Kornienko[1]*

[1]Institute of Inorganic Chemistry, University of Bonn, Gerhard-Domagk-Strasse 1, 53121 Bonn, Germany.
[2]University of Strasbourg, CNRS, ISIS UMR 7006, F-67000 Strasbourg, France

Correspondence to:: nkornien@uni-bonn.de

Experimental Section/Methods

Chemicals and Materials. 4,4'-bipyridyl (BPY) (99.97%), copper glycinate ($Cu(NH_2CH_2COO)_2$) (98.55%) 2,5-diformylfuran (DFF) (98%), 5-hydroxymethyl-2-furancarboxylic acid (HMFCA) (99.85%) and 5-formyl-2-furancarboxylic acid (FFCA) (98%) were purchased from BLD Pharmatech Ltd.. Copper acetate ($Cu(CH_3COO)_2$) (98%) was purchased from Alfa Aesar GmbH&Co KG. Glycerol (C3O3H8) (99%), furfural (98%), furfuryl alcohol (98%) and copper trifluoroacetate ($Cu(CF_3COO)_2$) (95%) was purchased from Thermo Scientific Chemicals. Methanol (CH_3OH) (99.8%) and diethyl ether ($(CH_3CH_2)_2O$) (99.5%) were purchased from Fisher Chemical. Potassium hydroxide (85%) and 5-Hydroxymethyfurfural (HMF) (95%) were purchased from Carl Roth GmbH&CoKG. 2,5-furandicarboxylic acid (FDCA) (98%) was purchased from abcr GmbH. Toray cabon paper, TGP-H-60, 19*19 cm was purchased from Thermo Fisher Scientific. Water (18.2 MΩ·cm) used in all experiments was prepared by passing through an ultra-pure purification system. All chemicals were used without further purification.

Synthesis of $Cu(X)_2$BPY. The BPY (109 mg) was dissolved into methanol in the bottle A, and the copper acetate (200 mg) was dissolved into methanol in the bottle B, then added solution A into solution B drop by drop, stirred 24 h. The product was washed with diethyl ether for several times and dried under vacuum overnight to get **$Cu(OAc)_2$BPY**.

Analogously, **$Cu(TFA)_2$BPY** and **$Cu(Gly)_2$BPY** were prepared using the same method by introducing trifluoroacetate and glycinate as co-ligand, respectively.

Characterizations. Low-resolution energy dispersive X-ray spectroscopy was performed on a scanning electron microscope (SEM, Hitachi, S-4700). The X-ray diffraction (XRD) measurements were carried out using a Stoe STADI P Mythen with a Co radiation source (λ = 1.78896 nm) for 2θ angles from 5° to 90° at a speed of 5° min^{-1}. Transmission electron microscopy (TEM) imaging was performed using a JEOL1400 TEM at an acceleration voltage of 120 kV. Fourier transform infrared (FT-IR) spectra were recorded on a Spectrum Two Lita FT-IR spectrometer (PerkinElmer, Llantrisant, UK). XAFS data were collected in fluorescence mode on BL12C of Photon Factory in the Institute for Structure Materials Science, High Energy Accelerator Research Organization (KEK-IMSS-PF) (2.5 GeV, 450 mA). All L-edge XAS data were analyzed using the program Demeter. The energy calibration was according to the absorption edge of the Pt foil. The data of XANES and EXAFS were

analyzed by Athena, Artemis, and hama software. X-ray photoelectron spectroscopy (XPS) and Cu Auger spectra measurements were carried out with a Thermo Scientific K-Alpha X-ray photoelectron spectrometer with a basic chamber pressure of $\approx 10^{-9}$ mbar and an Al anode as the X-ray source (X-ray radiation of 1486 eV). Spot sizes of 400 μm and pass energies of 200.00 eV for wide energy scans and 10.00−20.00 eV for scans were used. All XPS spectra and Cu Auger spectra were calibrated using the C 1s peak at 284.8 eV as a reference.

Electrochemical infrared spectroscopy study. *In situ* attenuated total reflection IR (ATR-IR) measurements were carried out using a Bruker Vertex 80 FTIR spectrometer with an MCT detector, which is cooled by liquid nitrogen. The ATR-IR crystal (germanium) was sputtered with Au, obtaining a thin gold layer on top of the ATR crystal, which acted as the working electrode. ART-IR measurements were carried out using an optical system integrated into the spectroscopic chamber. Absorbance spectra were obtained by calculating $A = -\log(I_1/I_2)$, where I_1 and I_2 represent the irradiation intensity of the incident and reflective beams, respectively.

Electrochemical measurements. All electrochemical measurements were conducted in a three-electrode system on an electrochemical workstation (Biological SP 50e) with a saturated Ag/AgCl as reference and a carbon rod as counter electrode, respectively. 0.1 M KOH with 10 mM HMF was used as electrolytes. The prepared $Cu(OAc)_2BPY$, $Cu(TFA)_2BPY$ or $Cu(Gly)_2BPY$ powder was dispersed into a mixed solution of water, ethanol, and Nafion in a volume ratio of 1.5:3.4:0.1, and the obtained catalyst liquid was then drop-dried onto the carbon paper (CP) to obtain $Cu(OAc)_2BPY$/CP, $Cu(TFA)_2BPY$/CP or $Cu(Gly)_2BPY$/CP, respectively, which were used as working electrodes. The catalyst loading amount was controlled at about 2 mg cm^{-2}. The flow cell was separated by FAA-3-PK-75 membrane and 1.0 M KOH + 50 mM HMF and 1.0 M KOH were used as anolyte and catholyte, respectively. All electrochemical data was converted to the reversible hydrogen electrode (RHE), and the calibration was performed in the hydrogen-saturated electrolyte solution with a platinum gauze electrode as the work electrode. Without specification, anodic polarization curves were iR-corrected according to the following equation: $E_{iR\text{-}corrected} = E - I*R_s$. Electrochemical impedance spectroscopy (EIS) tests were measured over a frequency range from 10^{-1} to 10^5 Hz with an AC amplitude of 10 mV in 1.0 M KOH and 10 mM HMF at 25 °C with the three-electrode cell system. In-situ electrochemical EIS was carried out from 10^5 to 10^{-2} Hz and amplitude was 10 mV. The electrochemical double layer capacitance (C_{dl}) was obtained by cyclic voltammetry (CV)

measurements at different scan rates of 20, 40, 60, 80, 100 and 120 mV s^{-1} in electrochemical cell. The potentials presented in this work were referenced to the reversible hydrogen electrode (V_{RHE}) in 0.1 M aqueous KOH (pH=13) and1.0 M aqueous KOH (pH=14) respectively using:

$$E\ (RHE) = E\ (Ag/AgCl) + 0.059 \times pH + 0.197\ (V)$$

The current density at each potential was measured for a constant potential for 300 s and the average current values were used to determine Tafel plots. The Tafel slope was calculated according to the Tafel equation as below:

$$\eta = b \times lg\ i + a$$

where η is the overpotential, i is the current density, a and b are defined as the Tafel constants.

Quantitative analysis of products. The liquid phase after reaction was collected to measure the concentrations using nuclear magnetic resonance (NMR).

The faradaic efficiency and selectivity were calculated according to equations, respectively.

$$Faradaic\ efficiency\ (\%) = 6 \times F \times n(FDCA\ generated)/total\ charge\ passed \times 100\%$$

$$FDCA\ selectivity\ (\%) = n(FDCA\ formed)/n(HMF\ consumed) \times 100\%$$

Where *F* is the Faraday constant (96485 C mol^{-1}), n is the mole number of reactants, calculated from the concentrations measured by NMR.

Theoretically, the oxidization of one HMF molecule to FDCA is a six-electron transfer process. Therefore, the total charge required for complete conversion of HMF to FDCA was calculated by the following equation:

$$6 \times (10\ mL) \times (10\ mM) \times (6.02 \times 10^{23}\ mol/L) \times (1.6 \times 10^{-19}\ C) = 57.8\ C$$

a

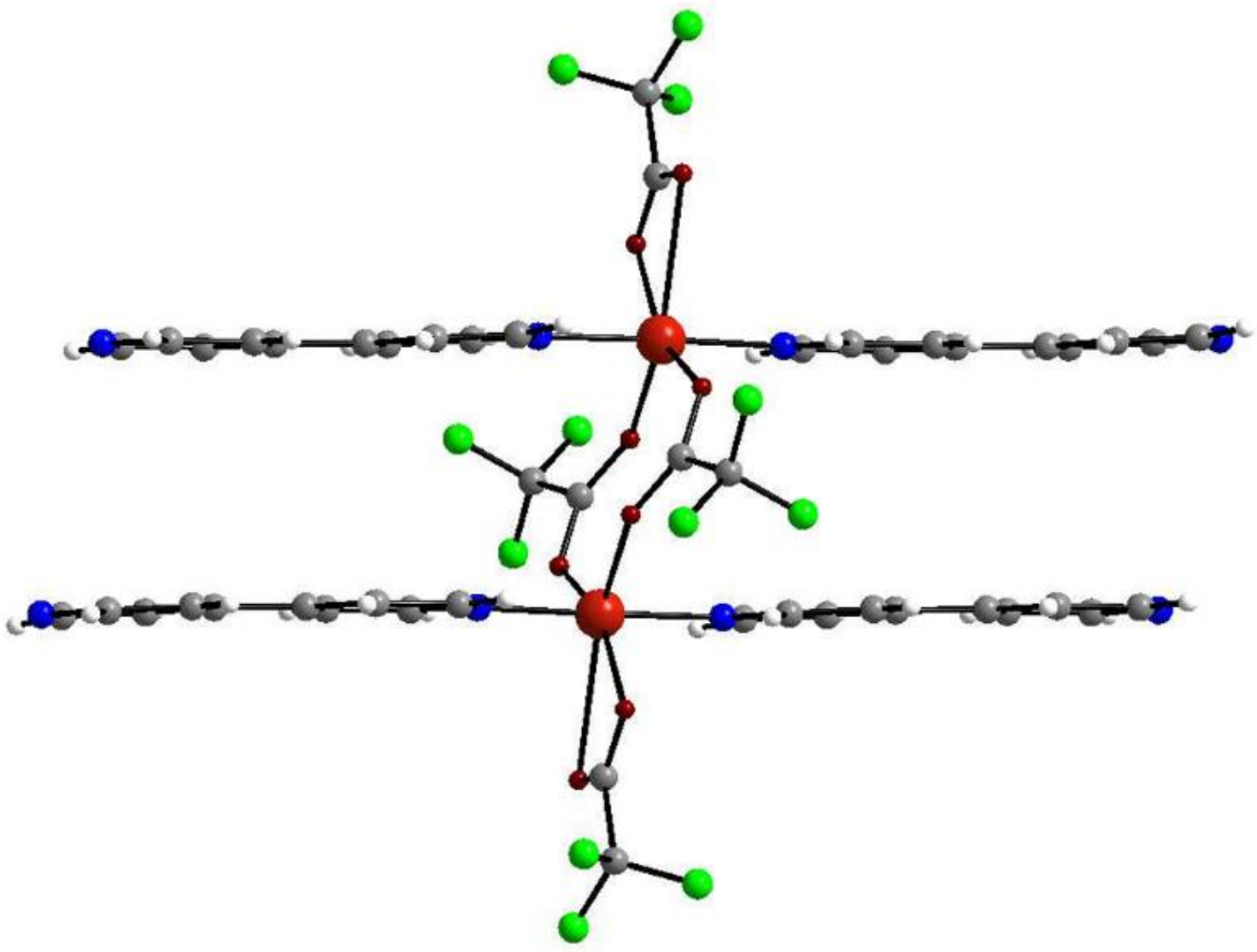

b

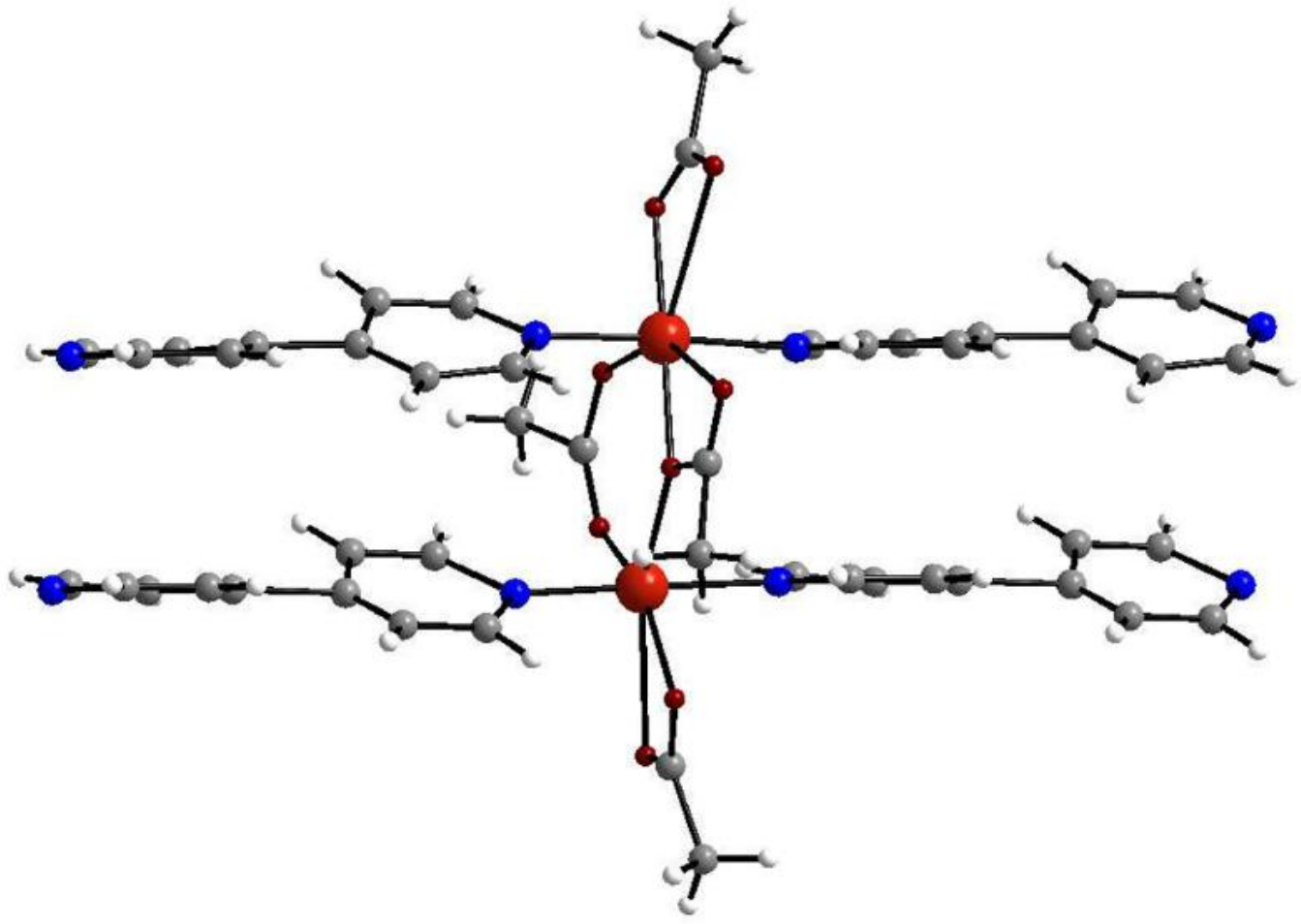

Figure S1 Crystal structures of (a) $Cu(TFA)_2BPY$ and (b) $Cu(OAc)_2BPY$ determined by single crystal X-ray diffraction.

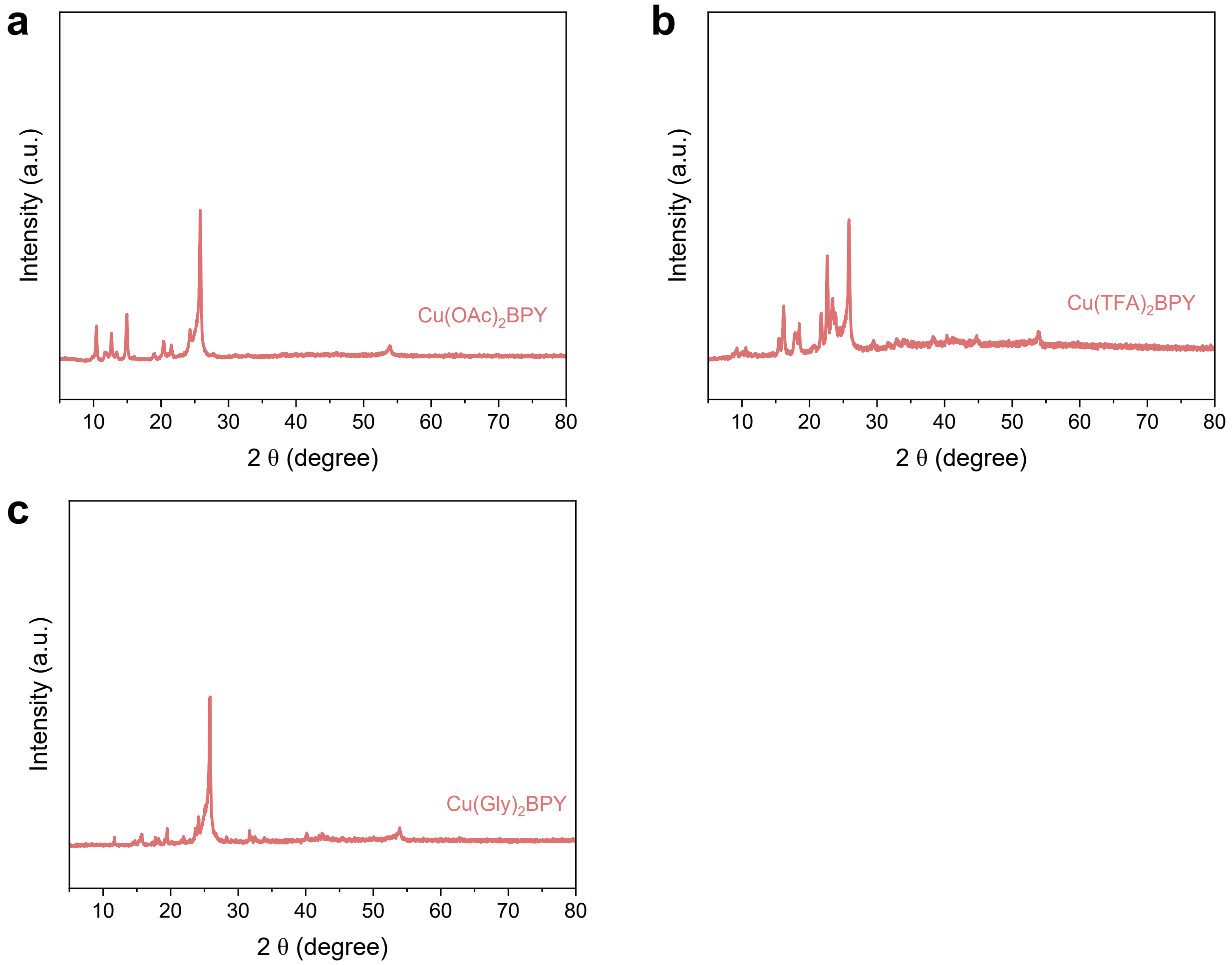

Figure S2 XRD patterns of (a) $Cu(OAc)_2BPY$, (b) $Cu(TFA)_2BPY$ and (c) $Cu(Gly)_2BPY$.

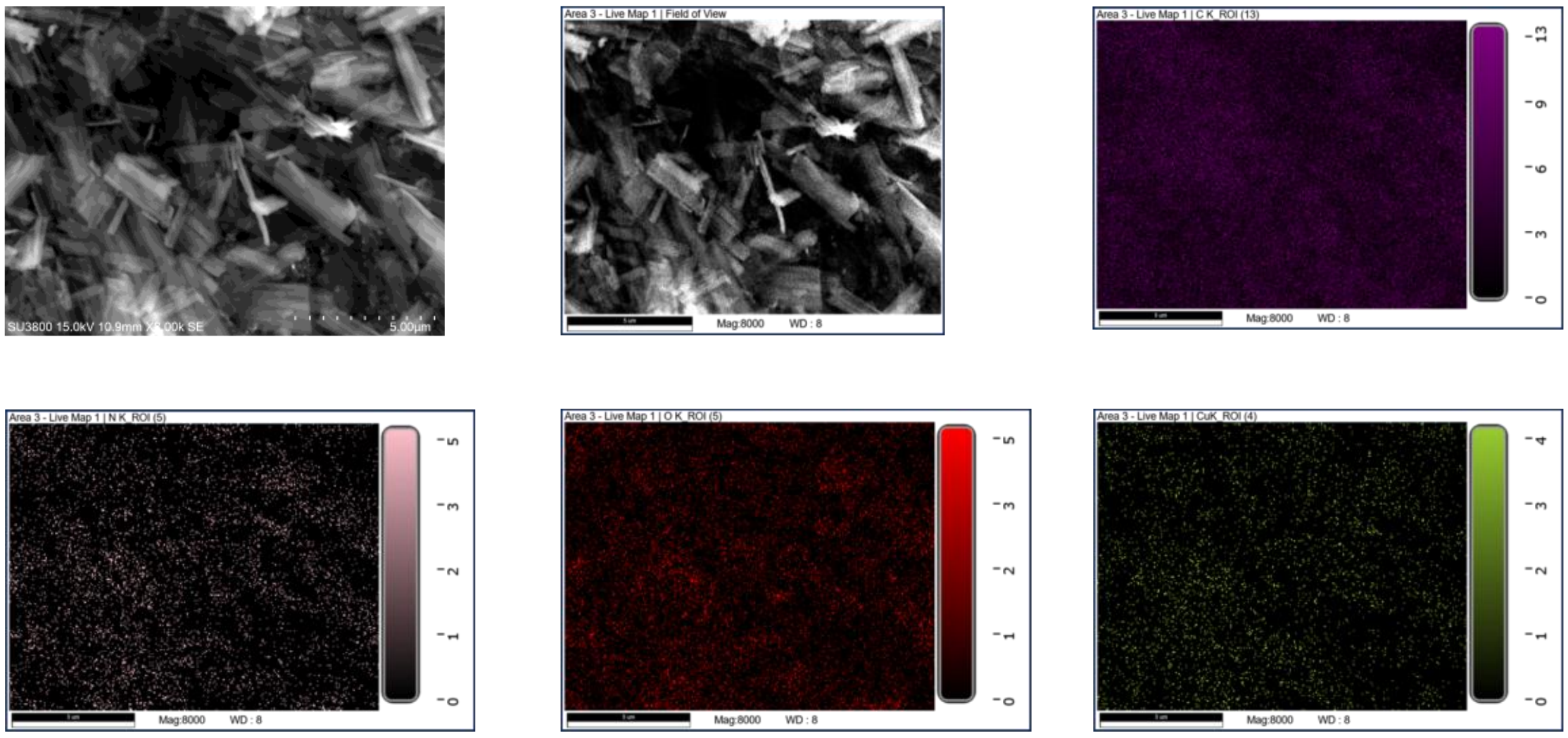

Figure S3 SEM image and elemental mappings of $Cu(OAc)_2BPY$.

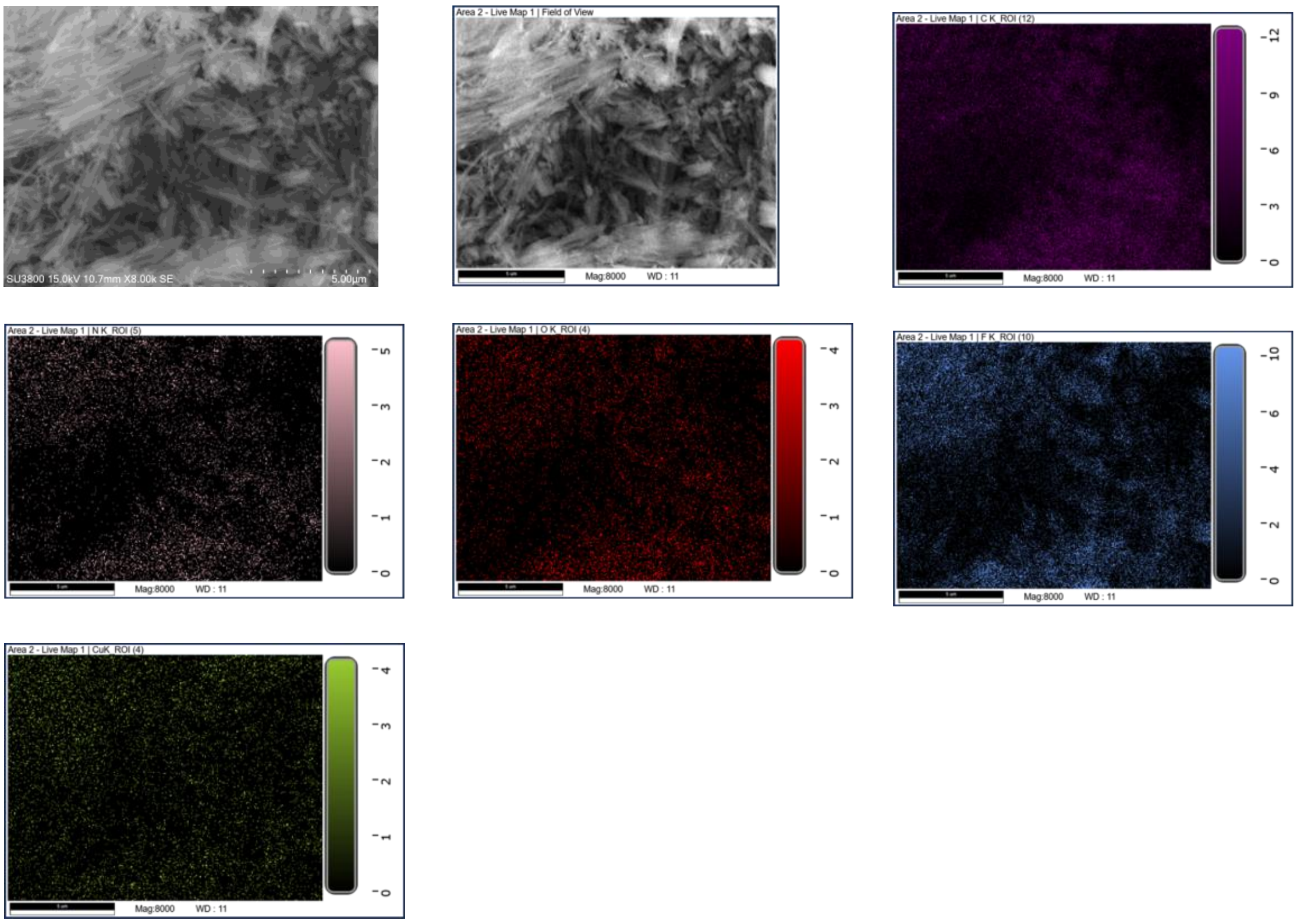

Figure S4 SEM image and elemental mappings of $Cu(TFA)_2BPY$.

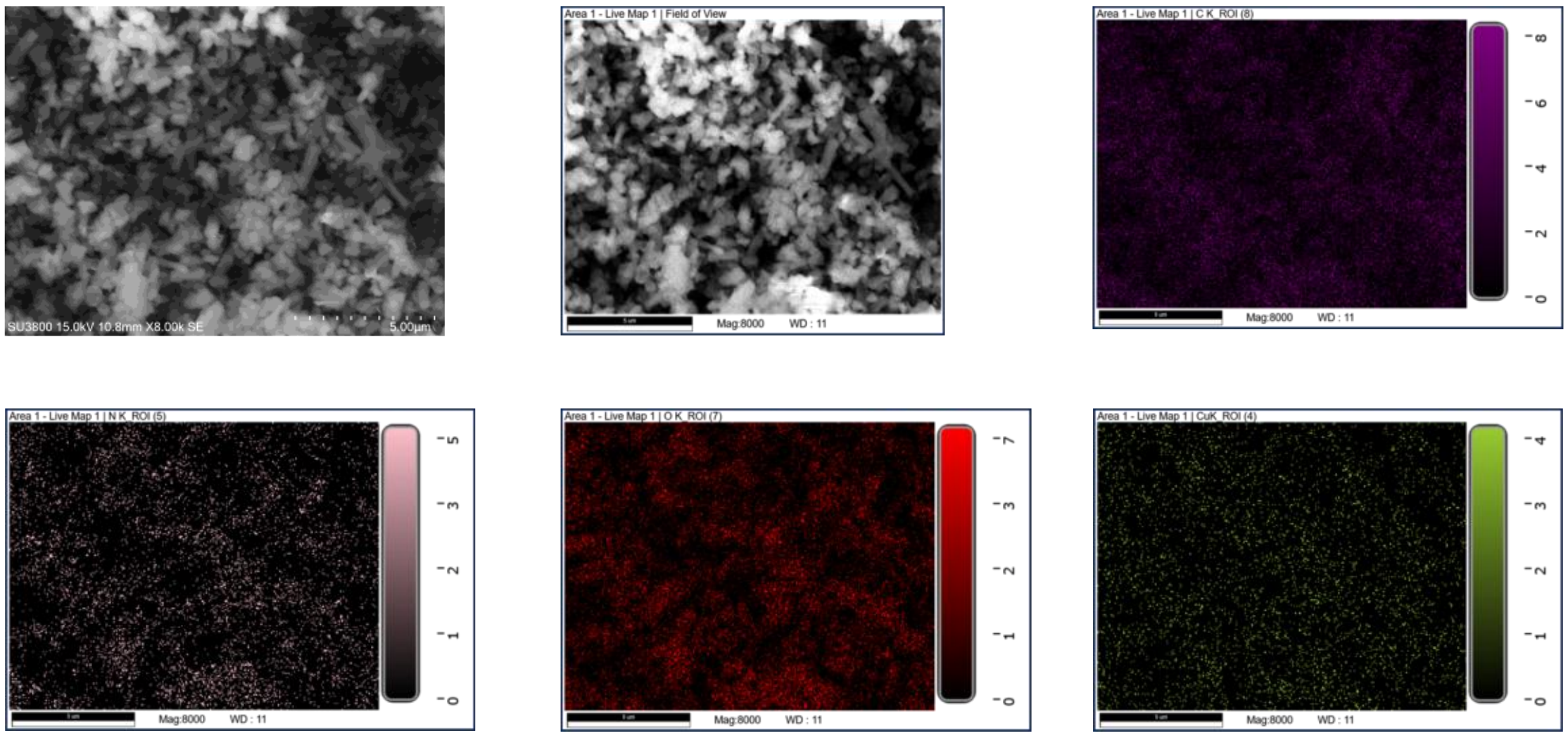

Figure S5 SEM image and elemental mappings of $Cu(Gly)_2BPY$.

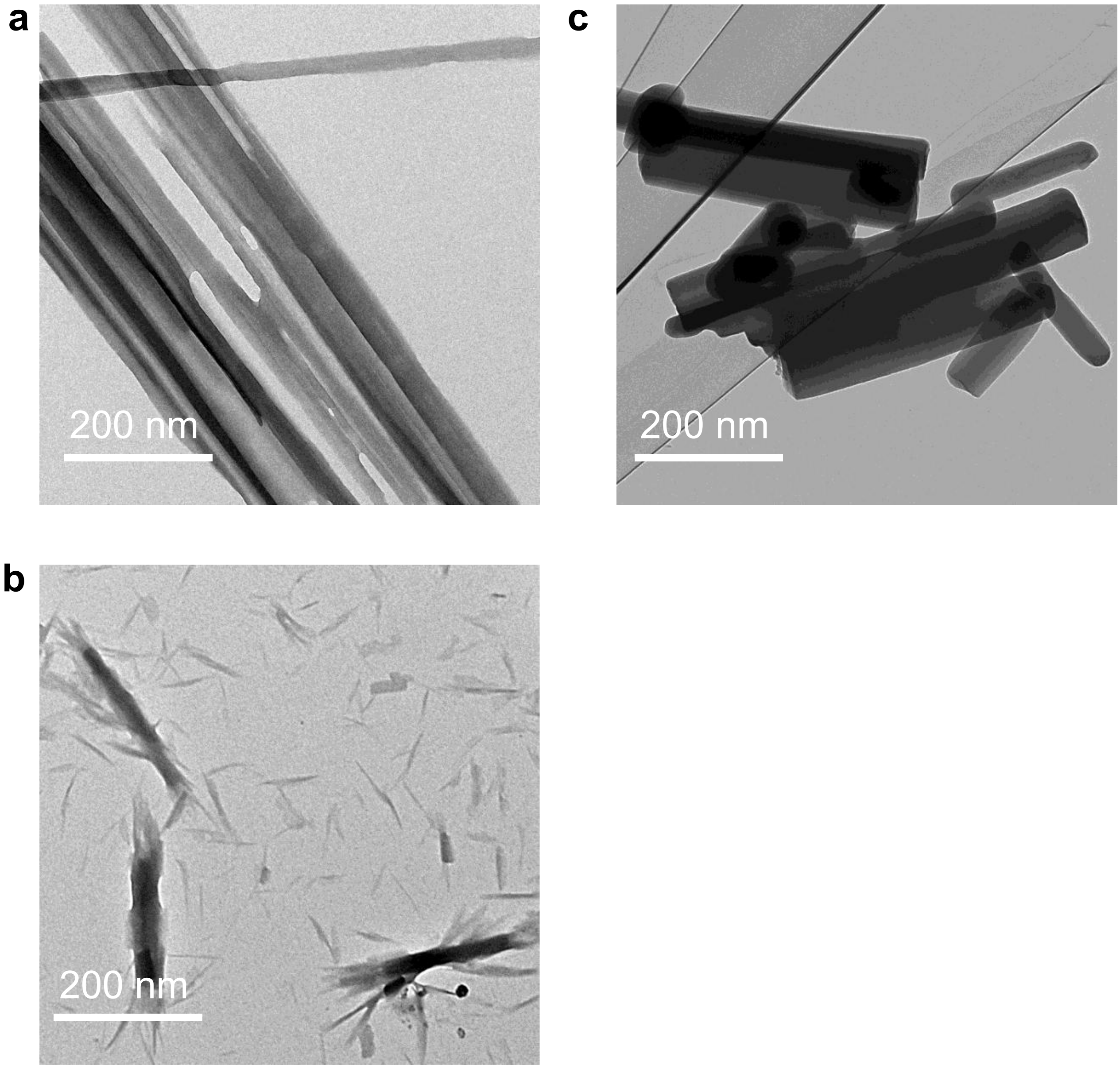

Figure S6 TEM images of (a) $Cu(OAc)_2BPY$, (b) $Cu(TFA)_2BPY$ and (c) $Cu(Gly)_2BPY$.

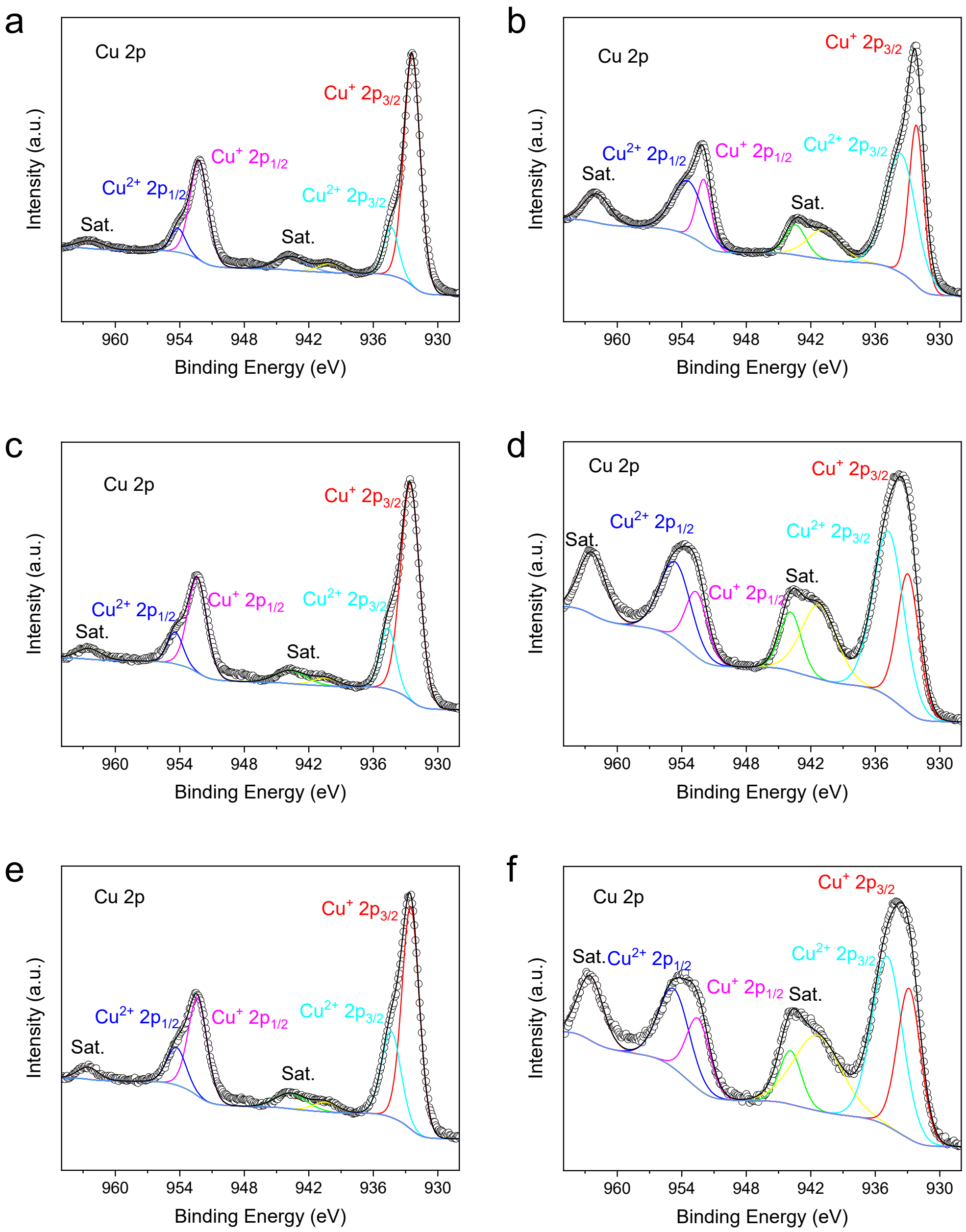

Figure S7 Cu 2p XPS spectra of $Cu(OAc)_2BPY$, $Cu(TFA)_2BPY$, and $Cu(Gly)_2BPY$ collected before HMFOR (a, c, e) and after HMFOR (b, d, f).

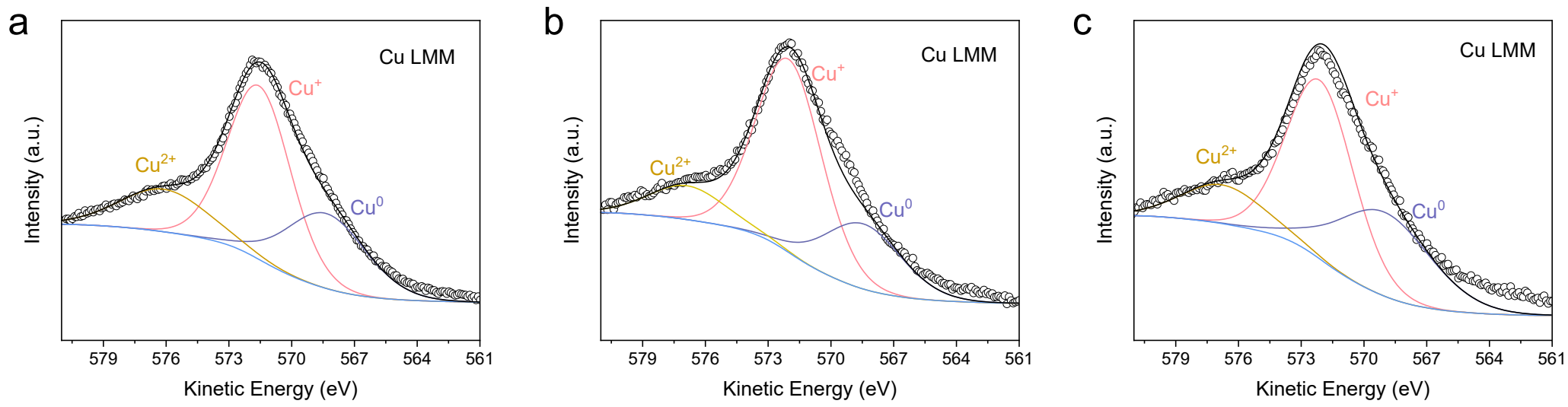

Figure S8 The Auger spectra of (a) $Cu(OAc)_2BPY$, (b) $Cu(TFA)_2BPY$, and (c) $Cu(Gly)_2BPY$.

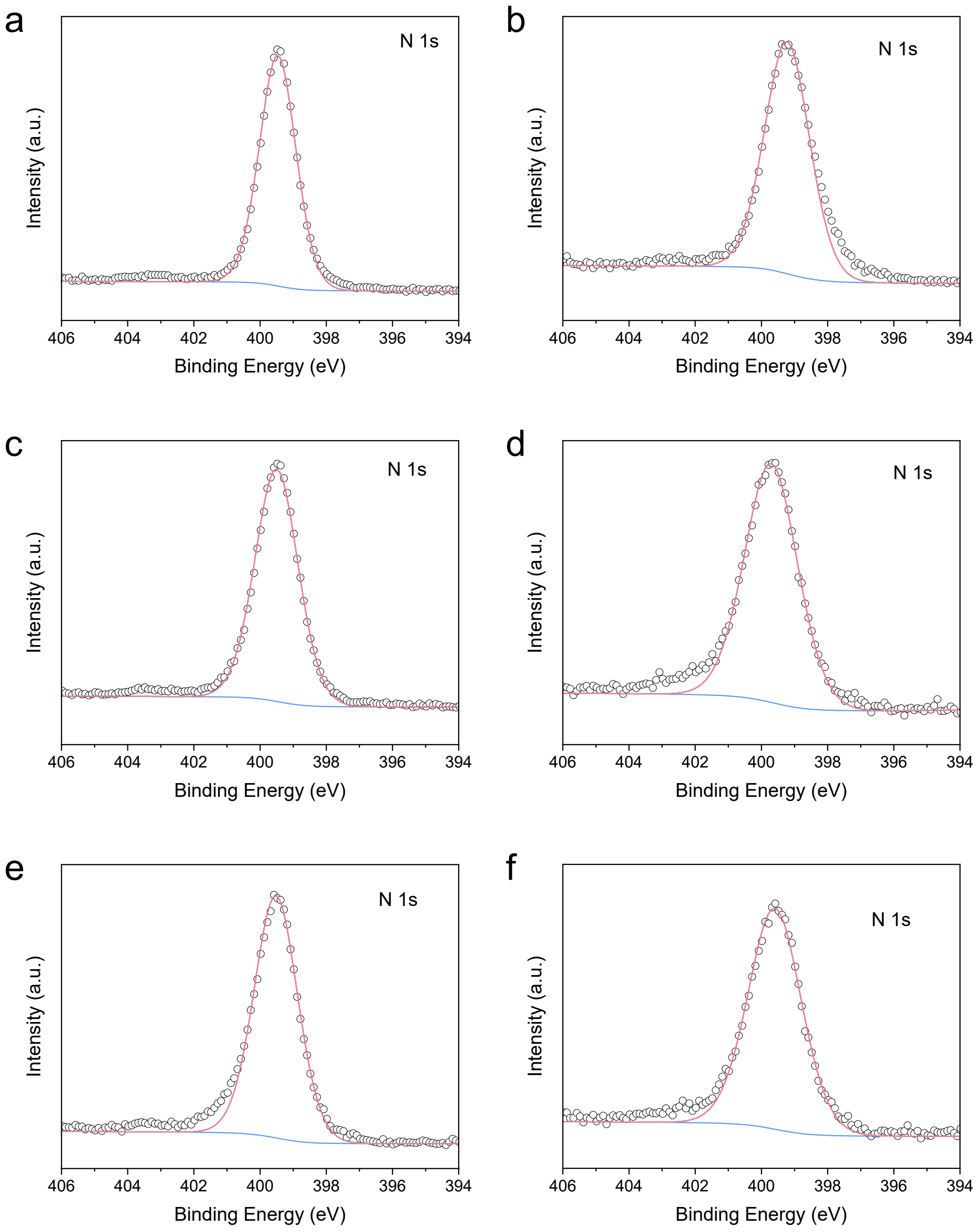

Figure S9 N 1s XPS spectra of $Cu(OAc)_2BPY$, $Cu(TFA)_2BPY$, and $Cu(Gly)_2BPY$ collected before HMFOR (a, c, e) and after HMFOR (b, d, f).

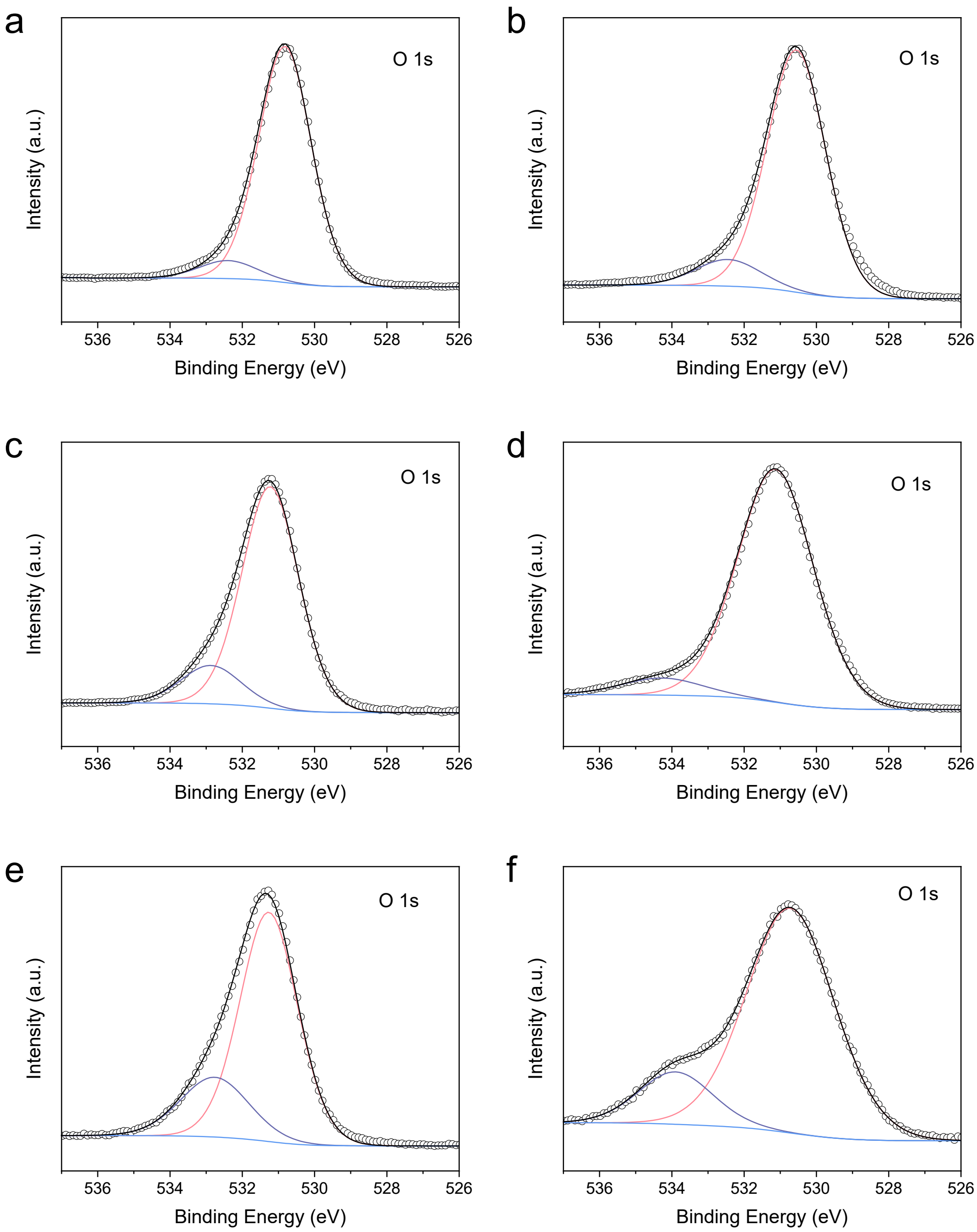

Figure S10 O 1s XPS spectra of $Cu(OAc)_2BPY$, $Cu(TFA)_2BPY$, and $Cu(Gly)_2BPY$ collected before HMFOR (a, c, e) and after HMFOR (b, d, f).

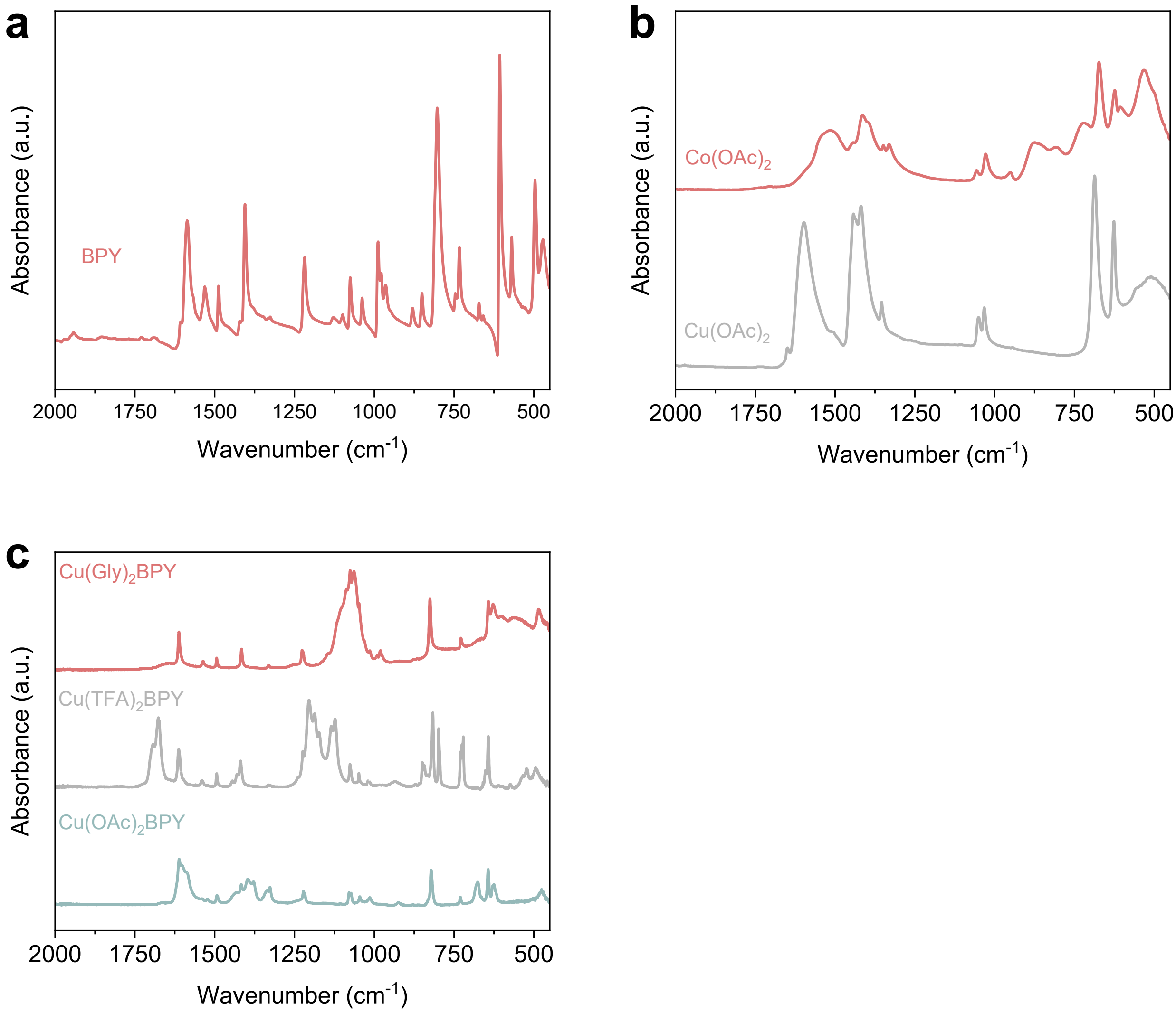

Figure S11 FT-IR spectra of (a) 4,4'-BPY, (b) $Cu(CH_3COO)_2$ and $Cu(CH_3COO)_2$ and (c) $Cu(OAc)_2BPY$, $Cu(TFA)_2BPY$ and $Cu(Gly)_2BPY$.

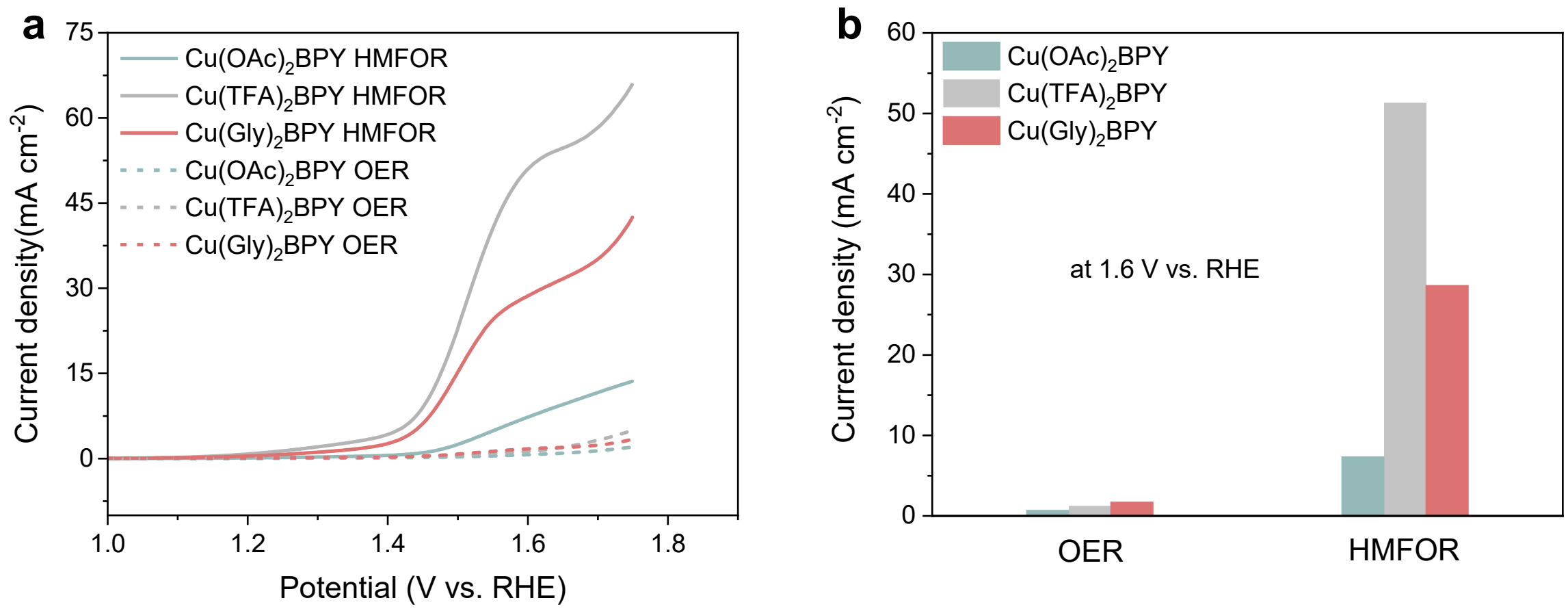

Figure S12 (a) LSV curves of $Cu(OAc)_2BPY$, $Cu(TFA)_2BPY$ and $Cu(Gly)_2BPY$ with and without 10 mM HMF. (b) Current density of these three catalysts at 1.6 V vs. RHE.

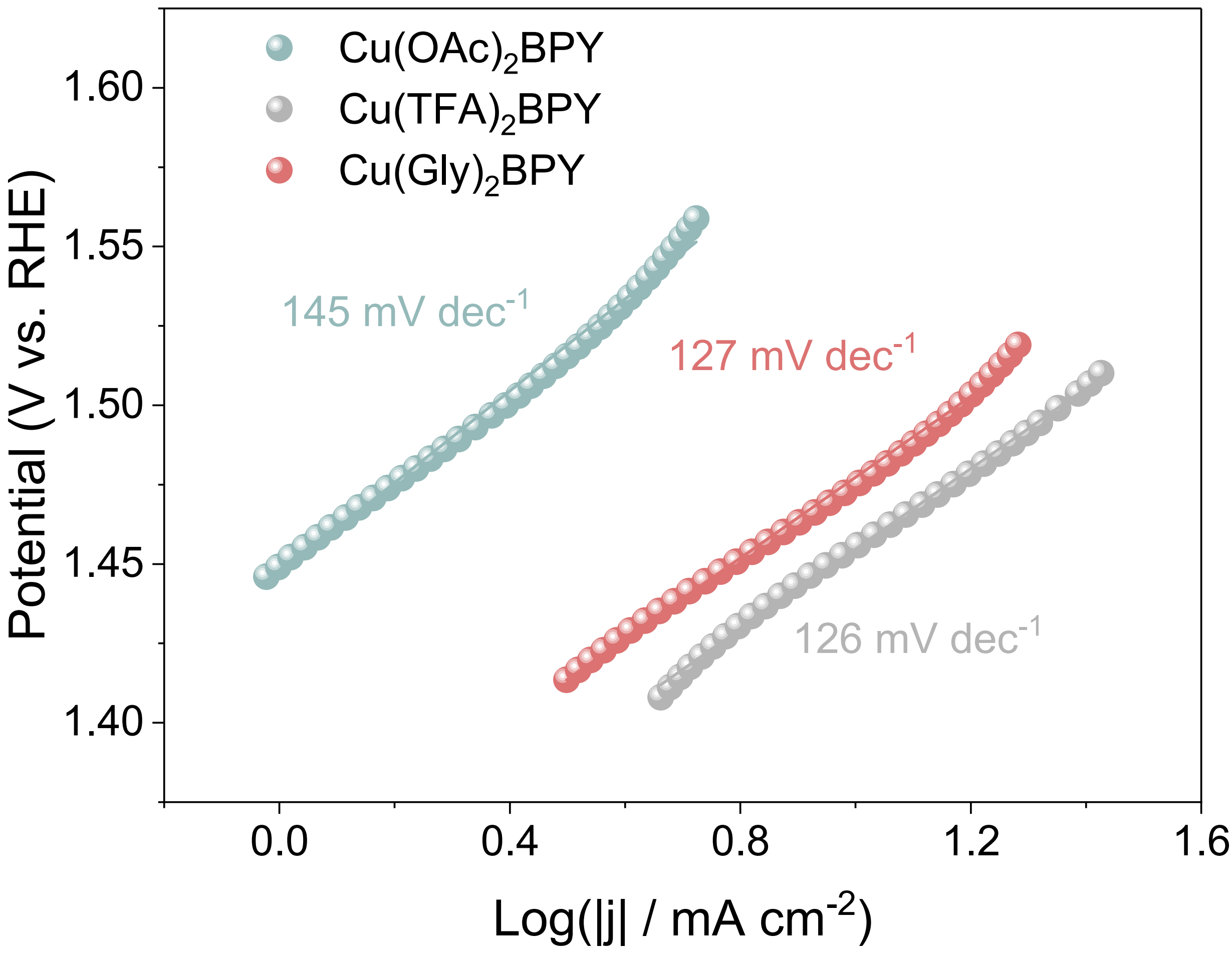

Figure S13 Tafel slopes of $Cu(OAc)_2BPY$, $Cu(TFA)_2BPY$ and $Cu(Gly)_2BPY$.

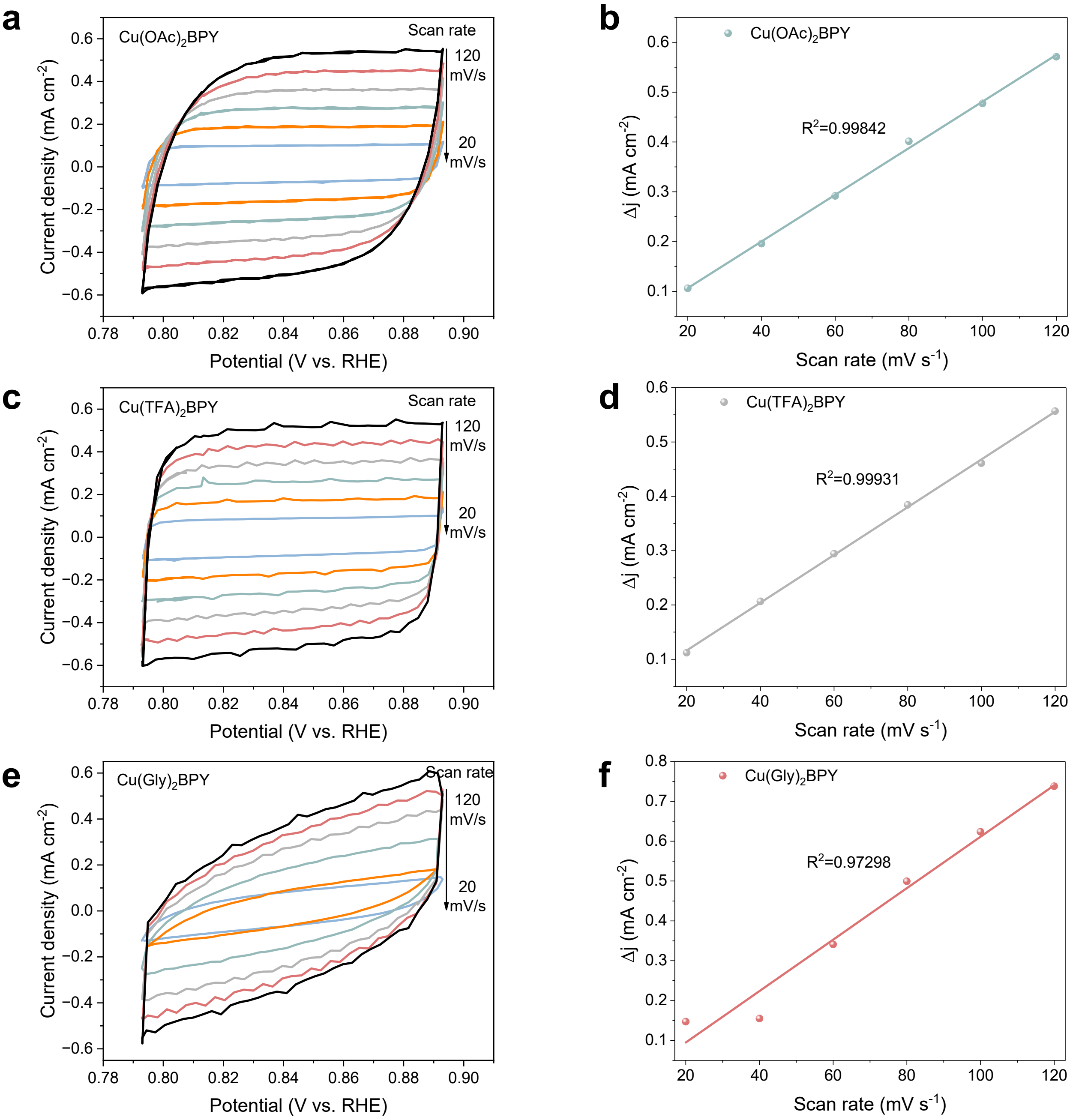

Figure S14 Electrochemical double-layer capacitance of (a and b) $Cu(OAc)_2BPY$, (c and d) $Cu(TFA)_2BPY$ and (e and f) $Cu(Gly)_2BPY$ for HMFOR.

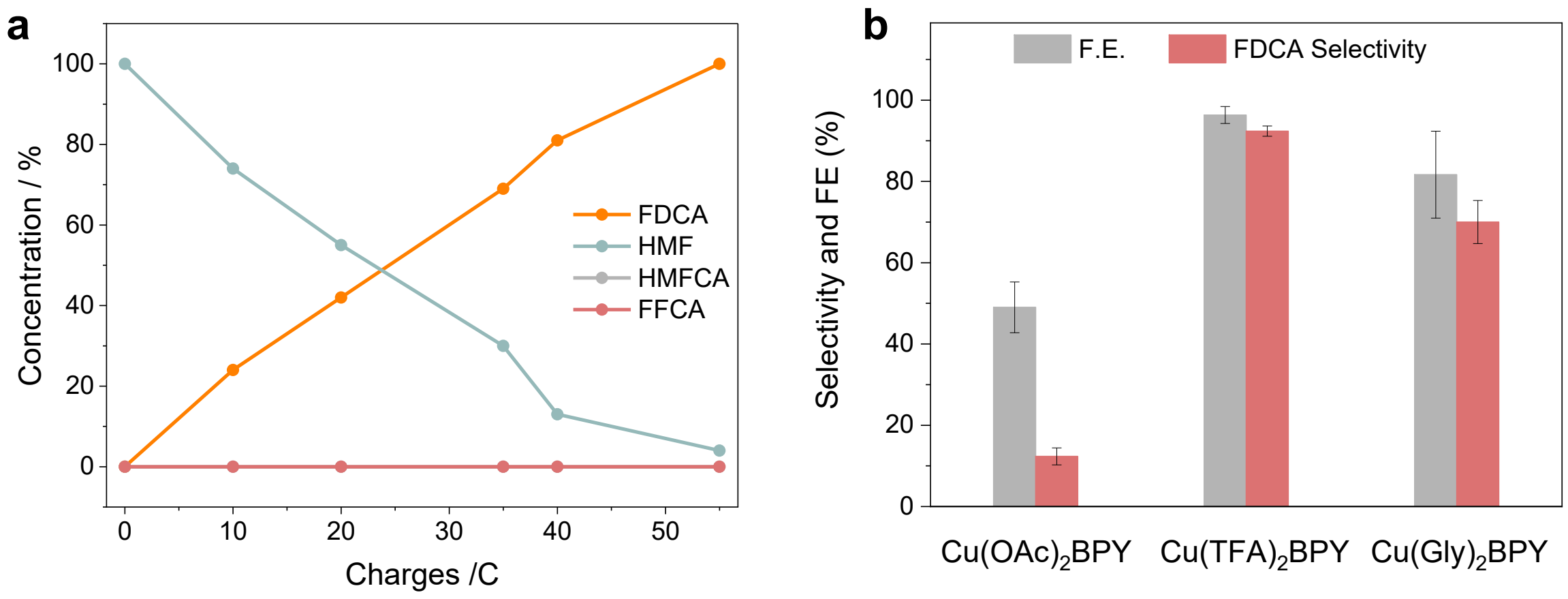

Figure S15 (a) Conversion of HMF and yields of products during HMFOR by using $Cu(TFA)_2BPY$. (b) Selectivity and FE of $Cu(OAc)_2BPY$, $Cu(TFA)_2BPY$ and $Cu(Gly)_2BPY$ at 1.6 V vs. RHE.

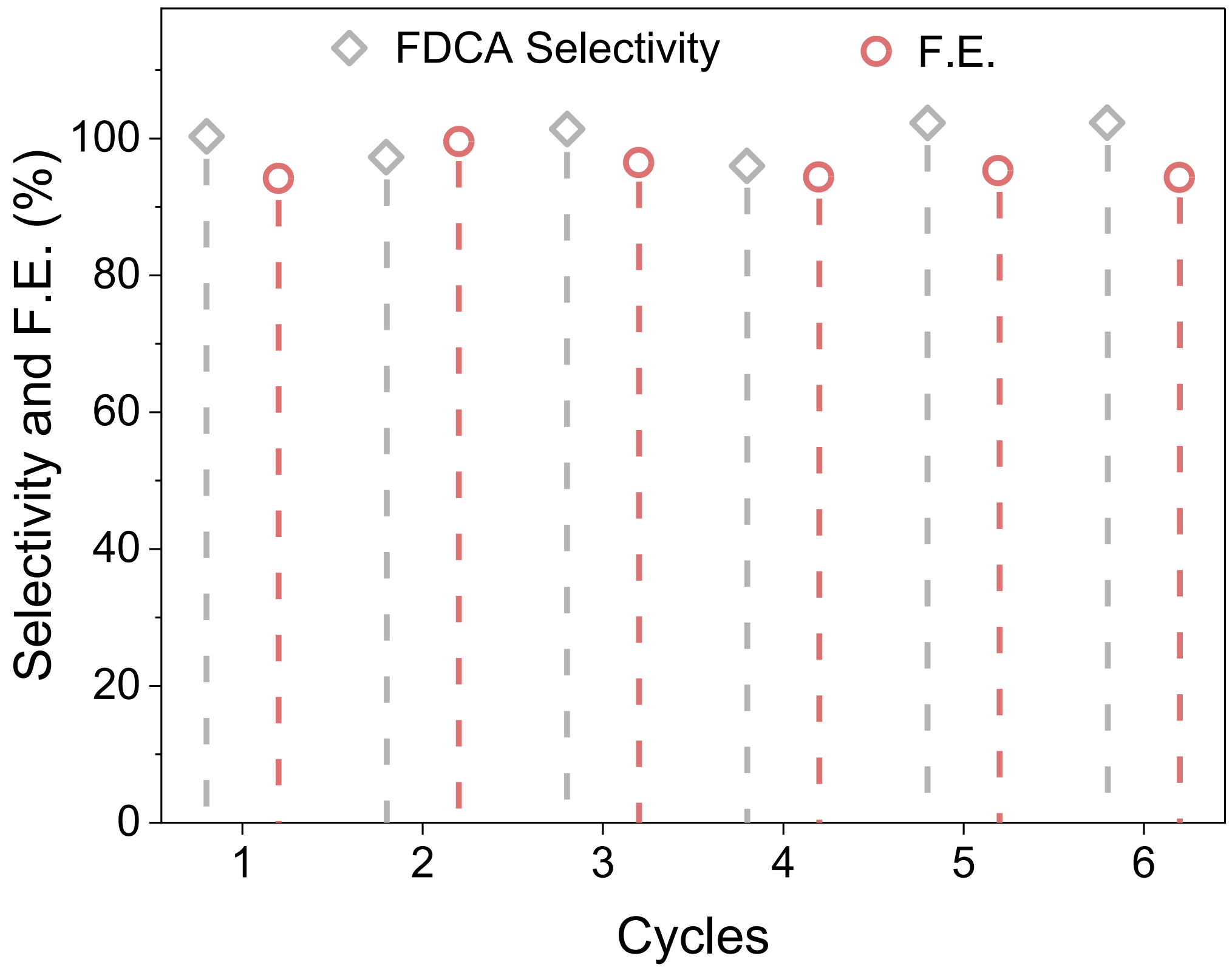

Figure S16 FDCA selectivity and FE toward HMFOR using $Cu(TFA)_2BPY$ under six consecutive electrolysis cycles.

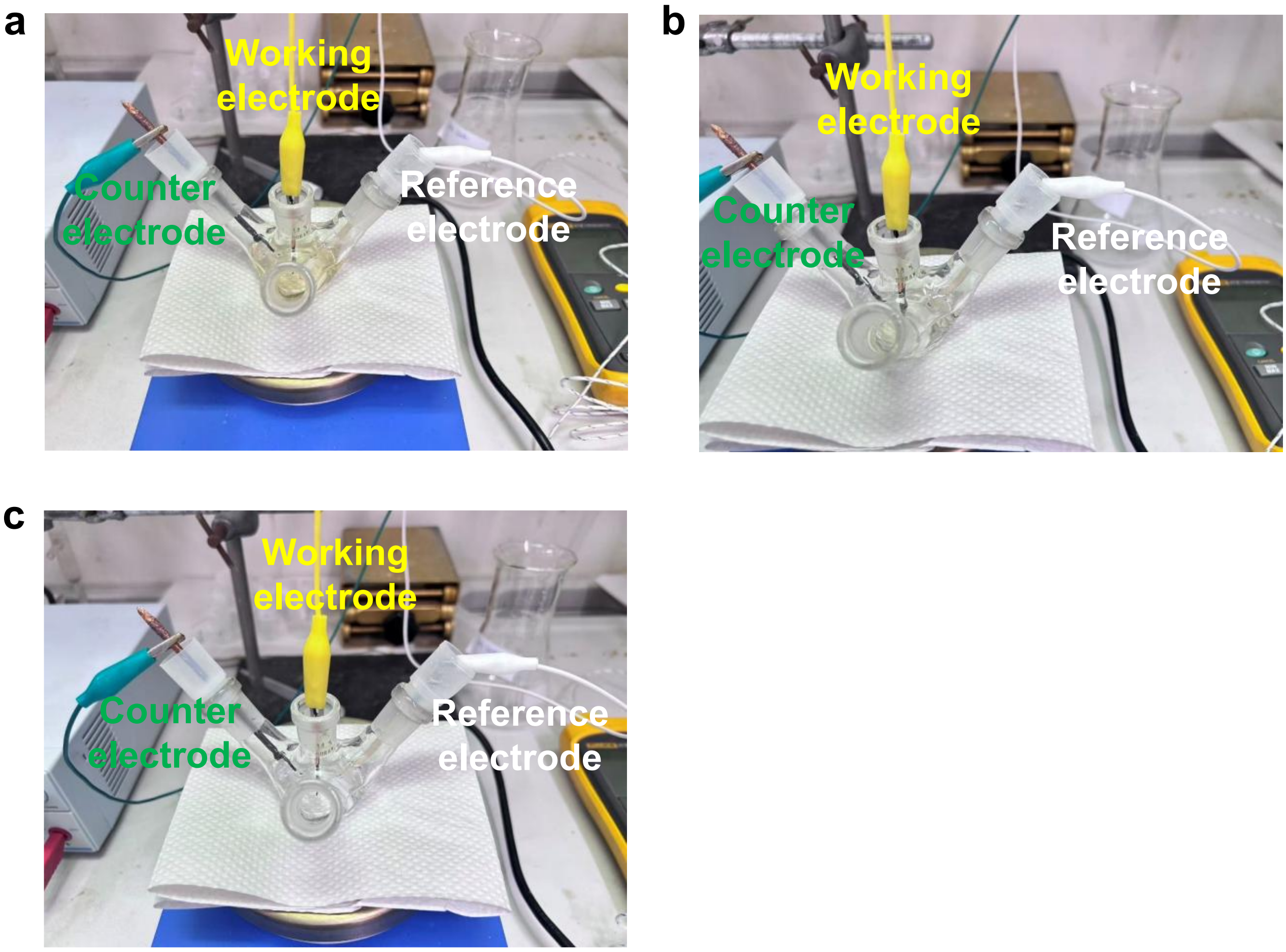

Figure S17 Photograph of three-electrode electrolytic cell for electrochemical HMF oxidation of (a) before electrolysis (b) during electrolysis, and (c) after electrolysis.

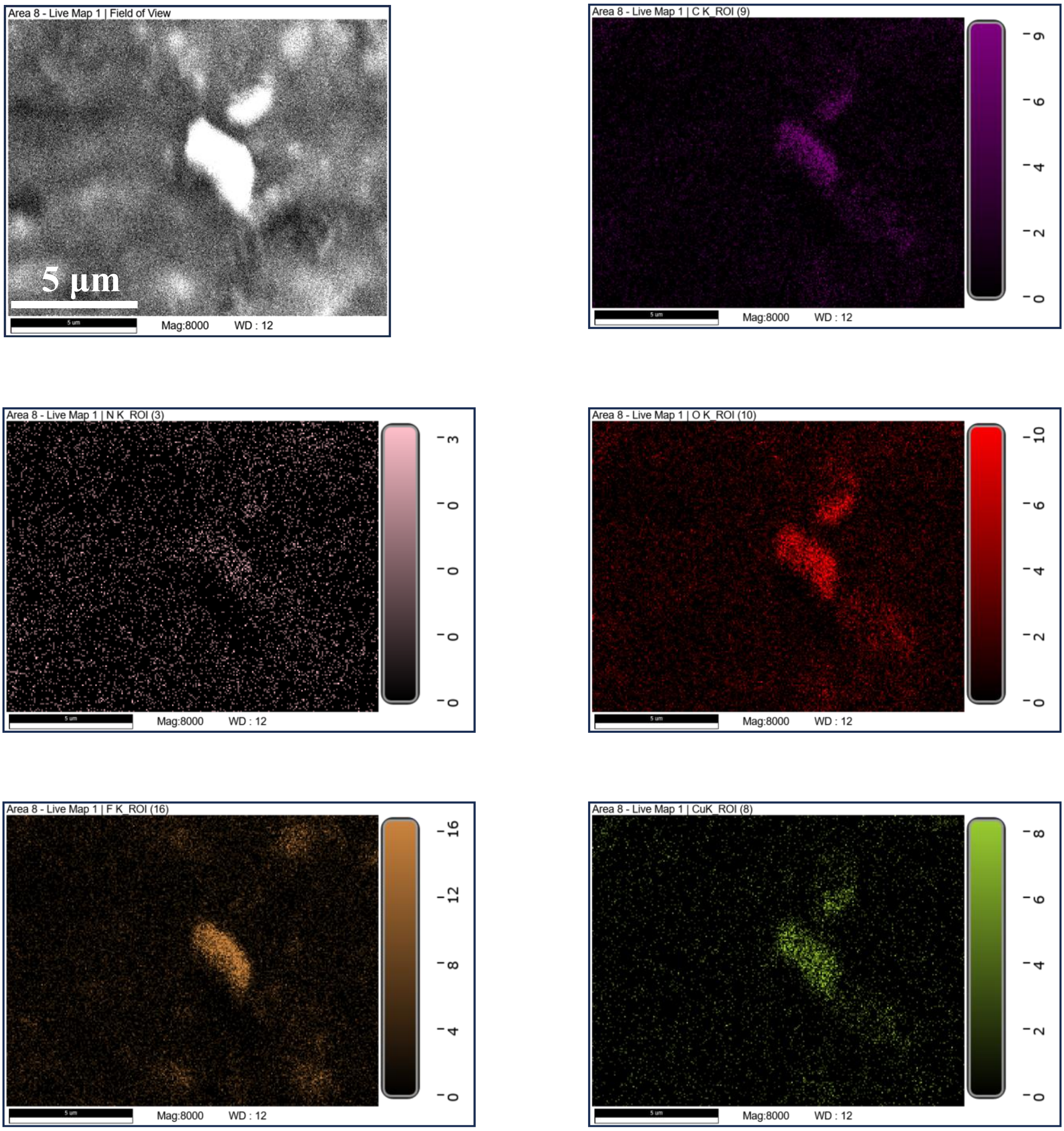

Figure S18 Post-reaction SEM and mapping images of $Cu(TFA)_2BPY$.

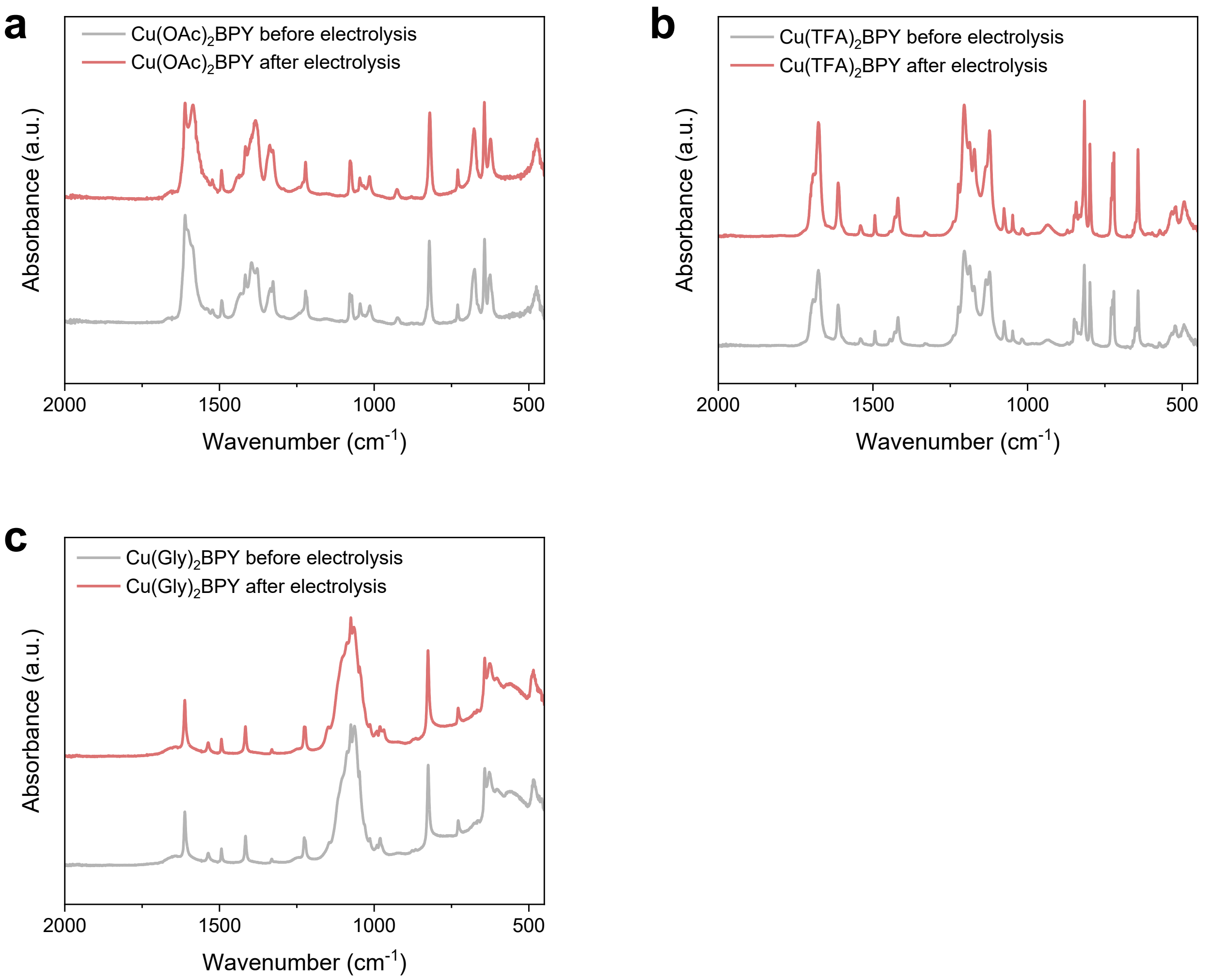

Figure S19 Before and after-reaction FT-IR spectra of (a) $Cu(OAc)_2BPY$ and (b) $Cu(TFA)_2BPY$ (c) $Cu(Gly)_2BPY$.

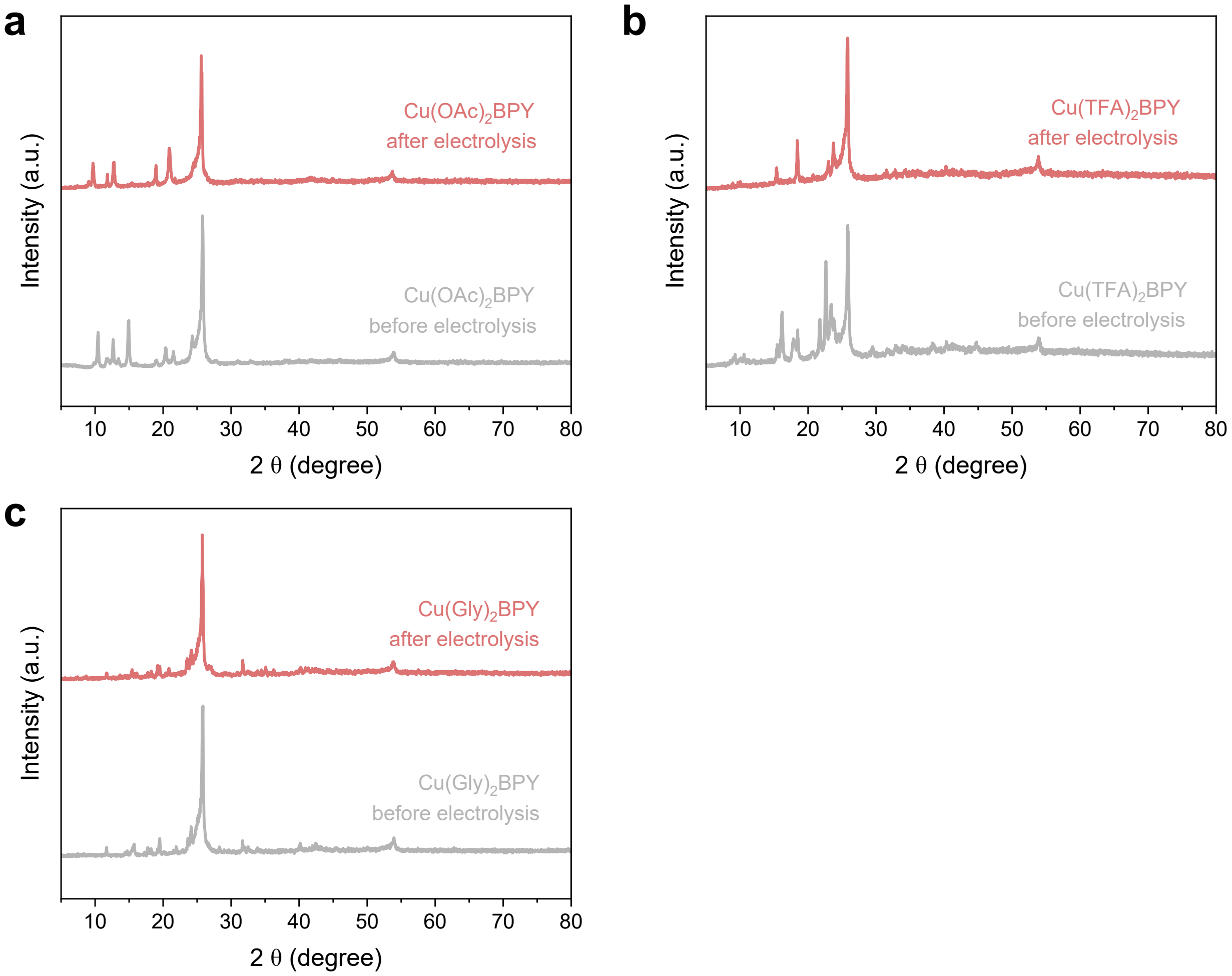

Figure S20 Before and after-reaction patterns of (a) $Cu(OAc)_2BPY$, (b) $Cu(TFA)_2BPY$ and (c) $Cu(Gly)_2BPY$

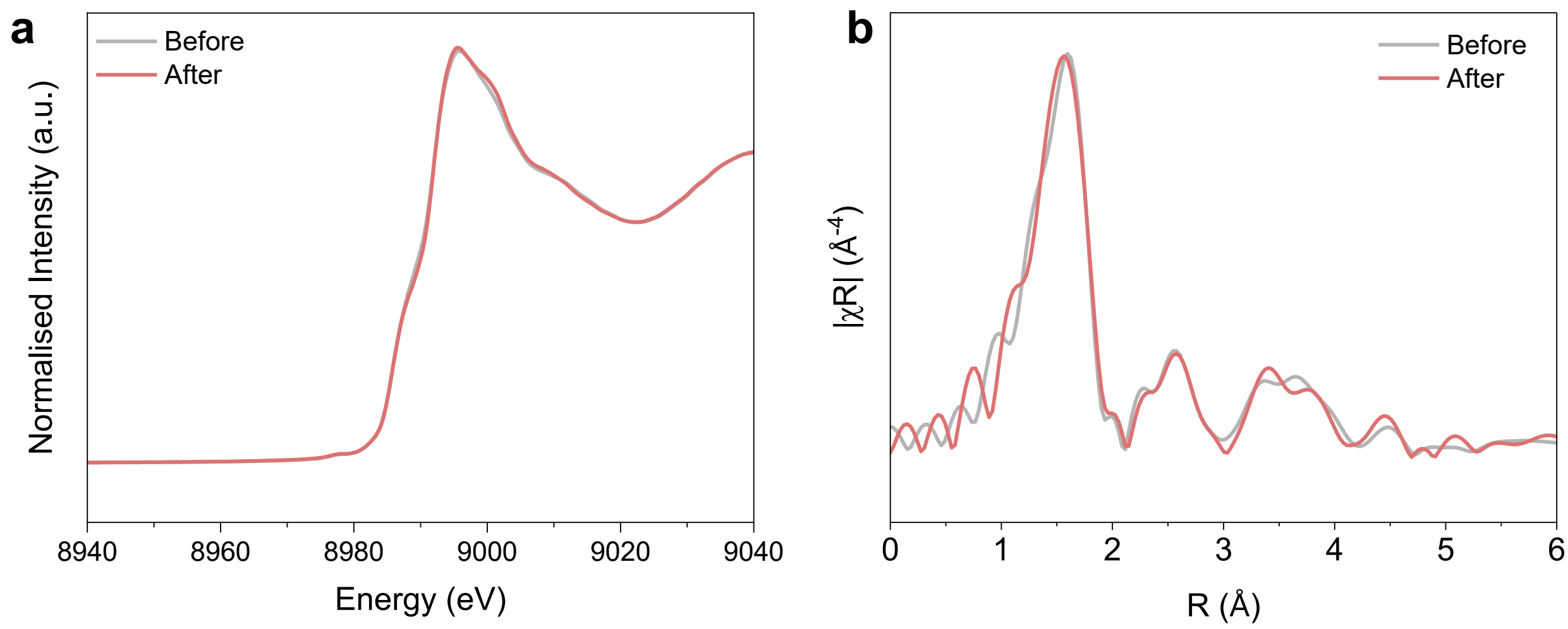

Figure S21 Before and after-reaction XAS spectra of $Cu(TFA)_2BPY$ of (a) K-edge and (b) R space.

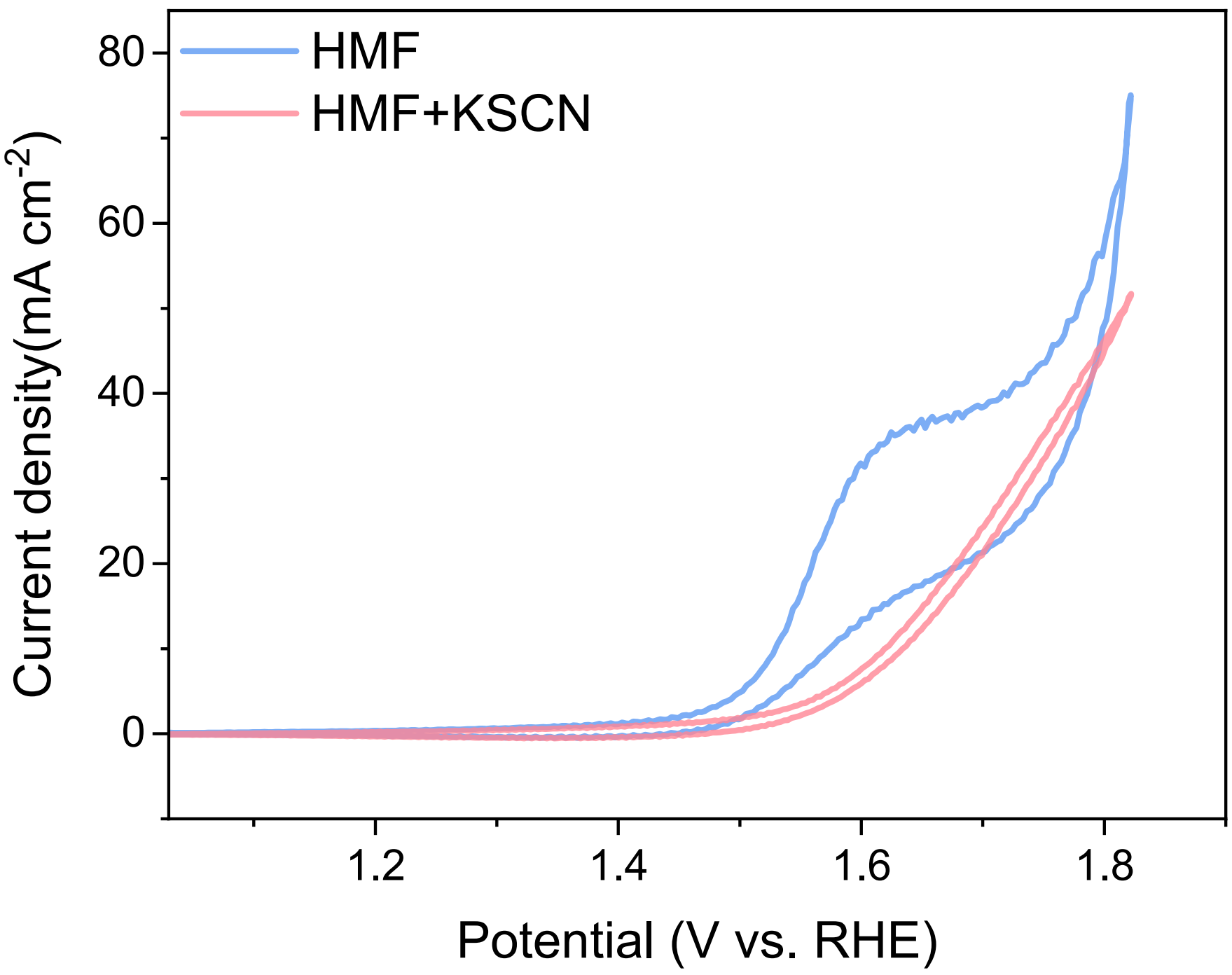

Figure S22. CV curves of $Cu(TFA)_2BPY$ recorded in HMF-containing electrolyte with and without KSCN.

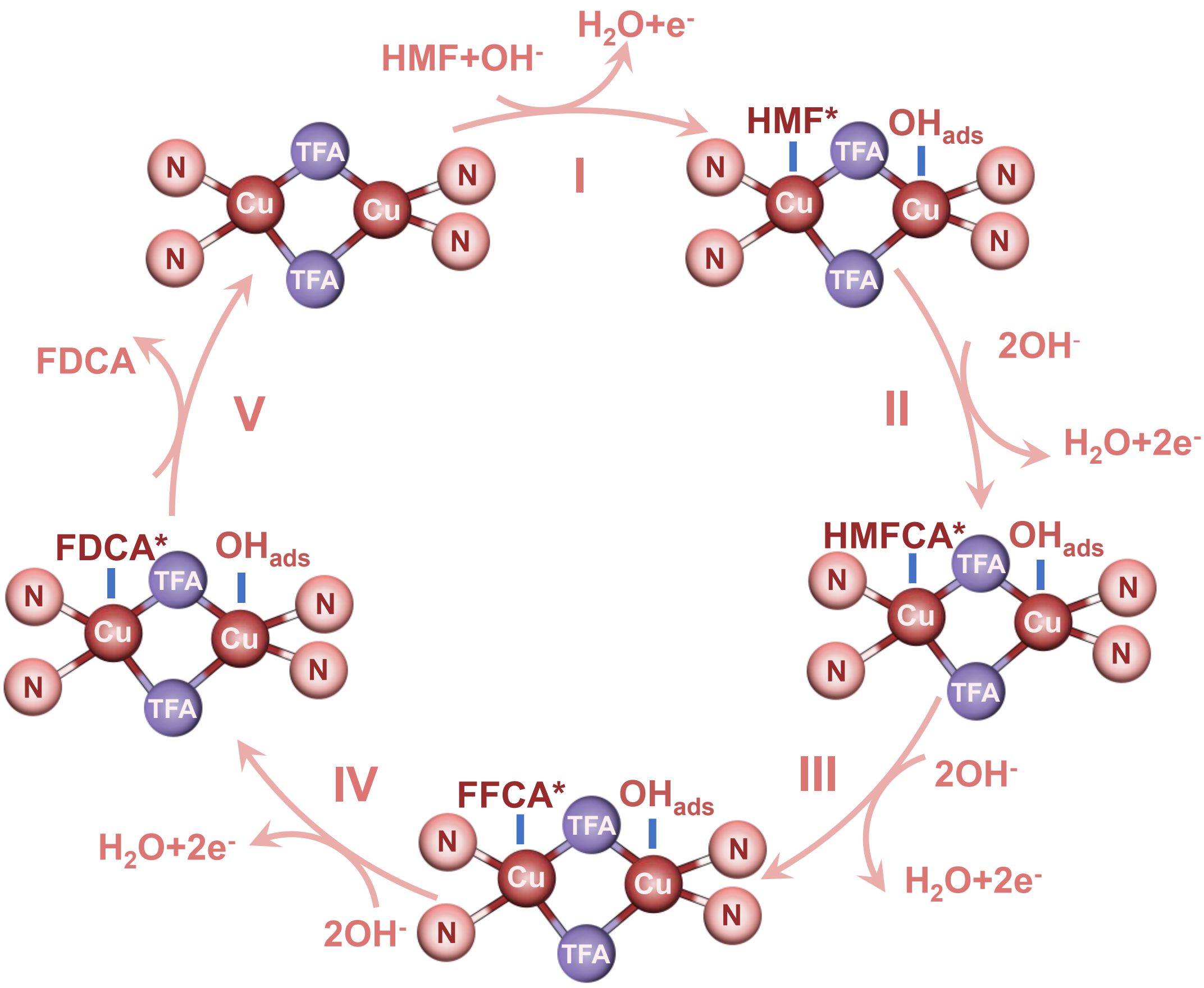

Figure S23 Schematic illustration of the HMFOR mechanism over $Cu(TFA)_2BPY$.

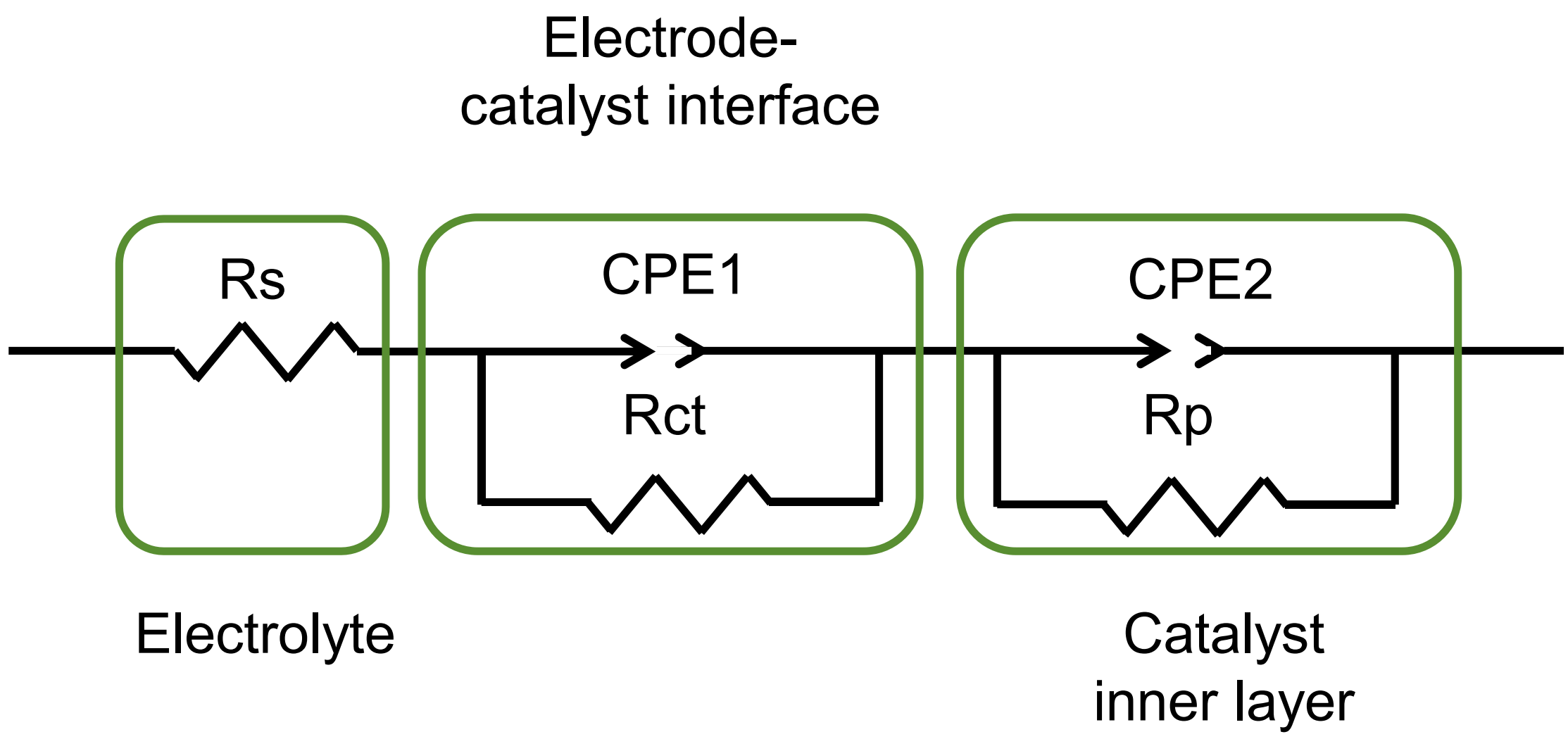

Figure S24 Simulated equivalent circuit of the catalysts during the reaction. Rs stands for the solution resistance, CPE1 represents double-layer constant phase angle element, Rct is related to the interfacial charge transfer rate, CPE2 and Rp are related to the dielectric properties and the resistance of the electrode inner film.

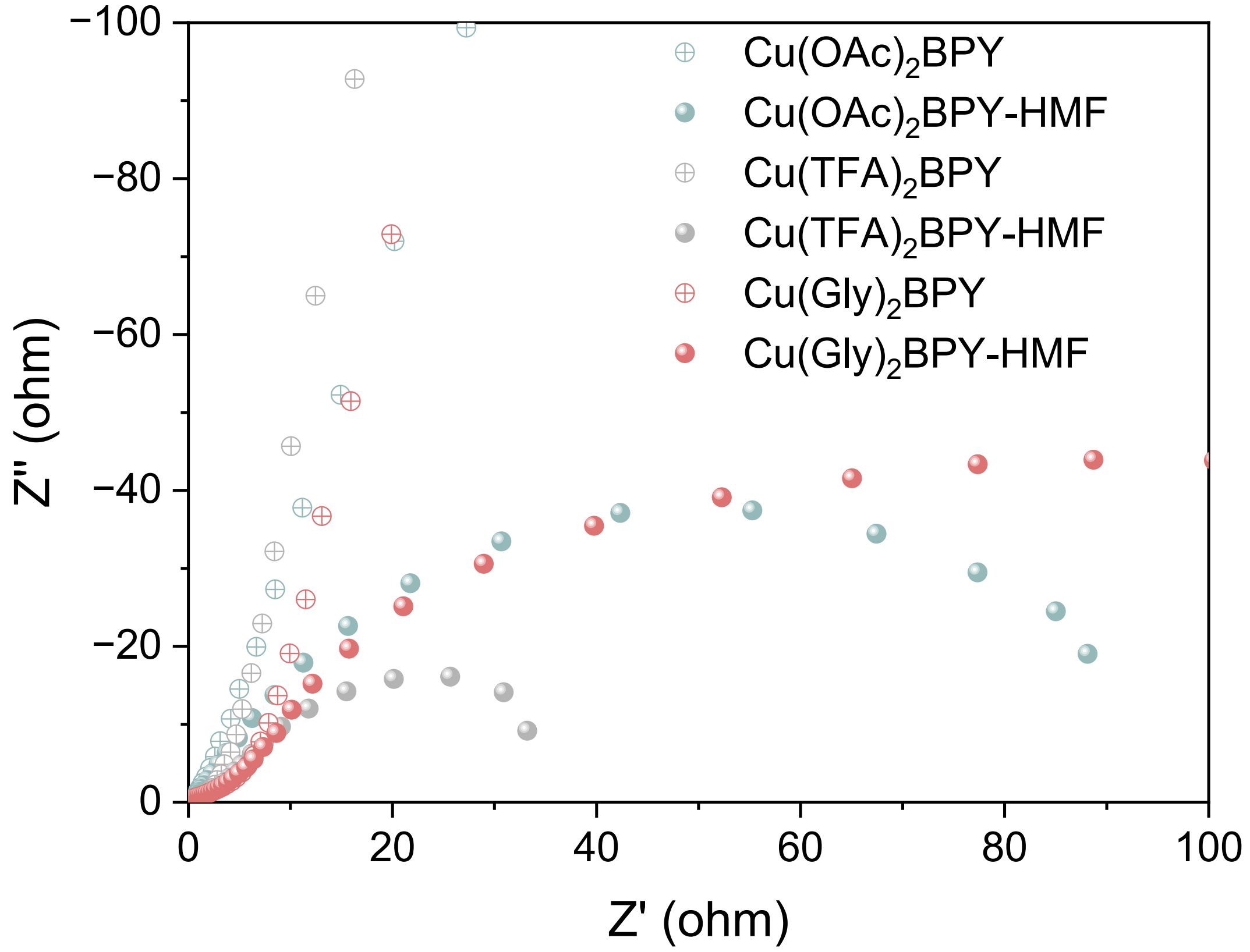

Figure S25 EIS Nyquist plots of $Cu(OAc)_2BPY$, $Cu(TFA)_2BPY$ and $Cu(Gly)_2BPY$ catalysts for OER and HMFOR.

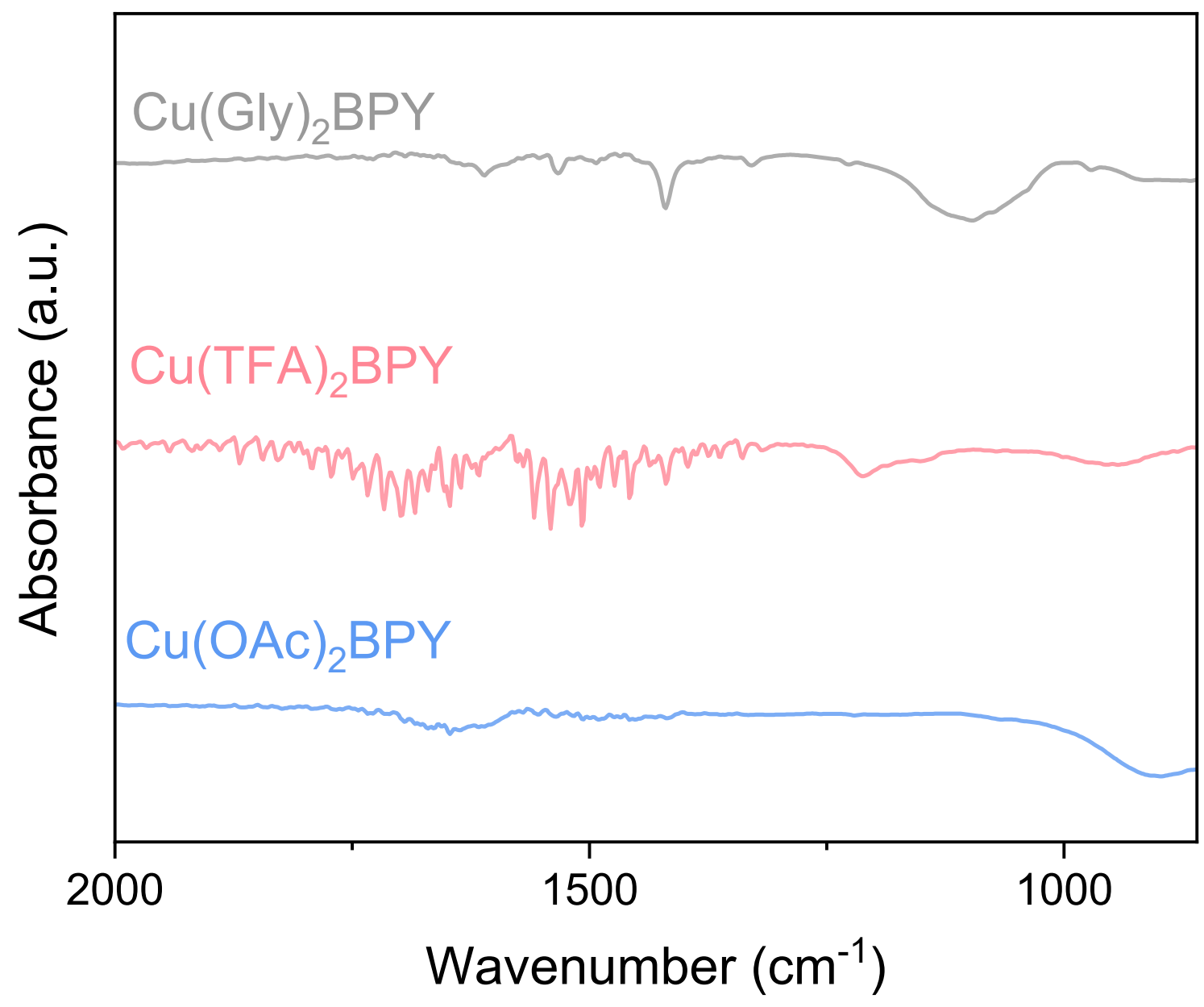

Figure S26 ATR-FTIR spectra of HMF adsorbed on Cu(OAc)$_2$BPY, Cu(TFA)$_2$BPY, and Cu(Gly)$_2$BPY.

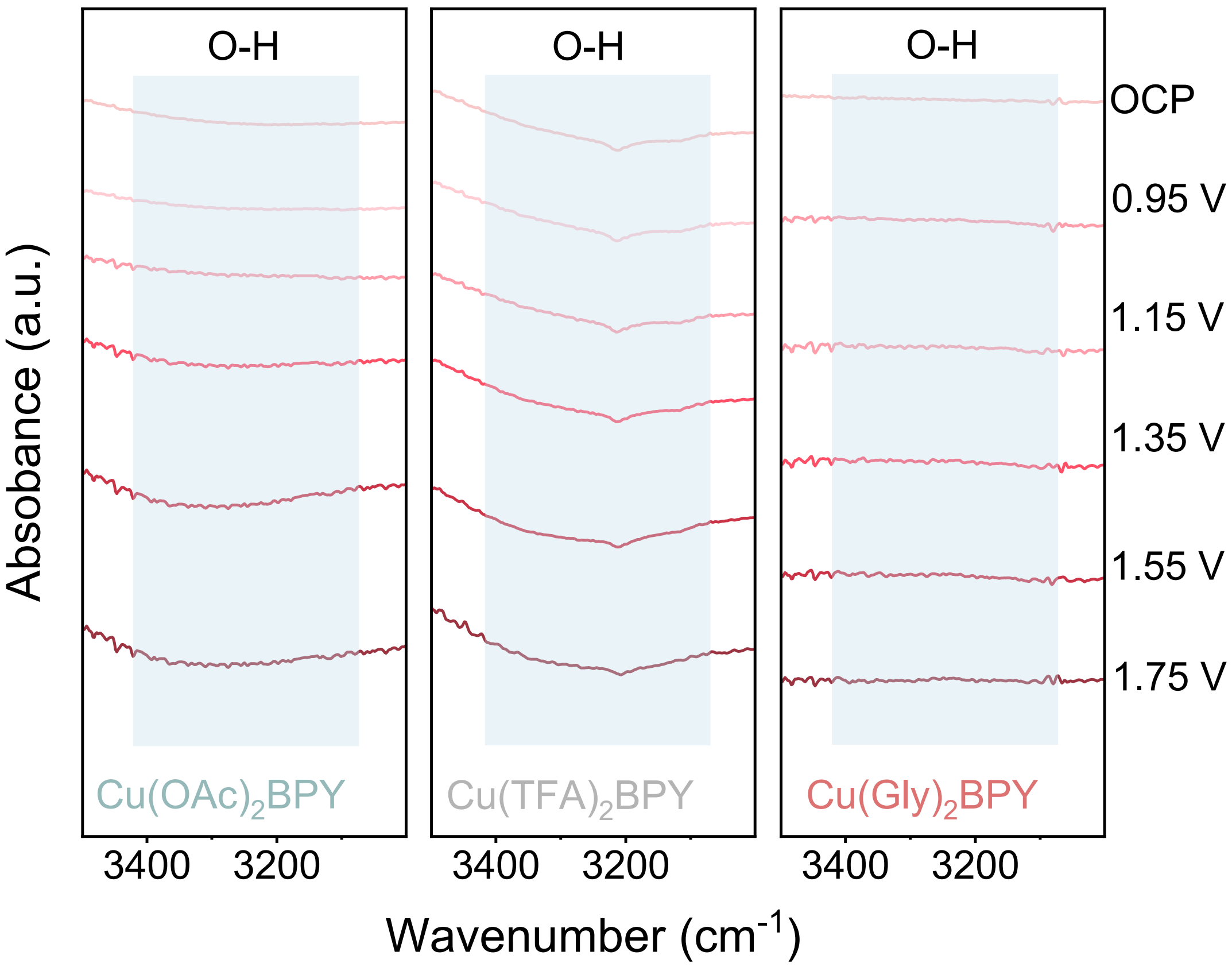

Figure S27 *In situ* ATR-FTIR spectra of $Cu(OAc)_2BPY$, $Cu(TFA)_2BPY$, and $Cu(Gly)_2BPY$ in high wavenumber regions.

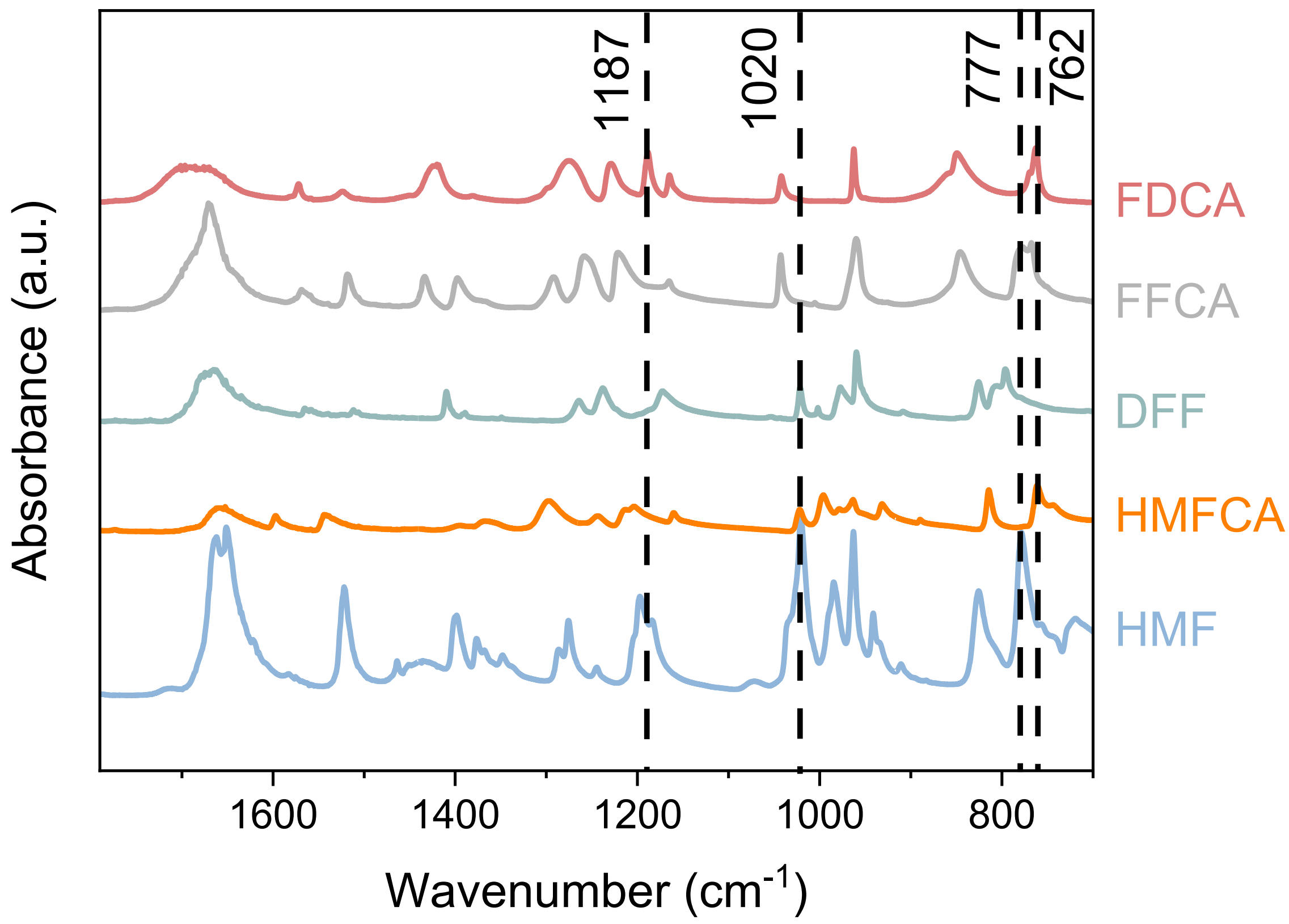

Figure S28 FT-IR spectra of potential products formed during the oxidation of HMF.

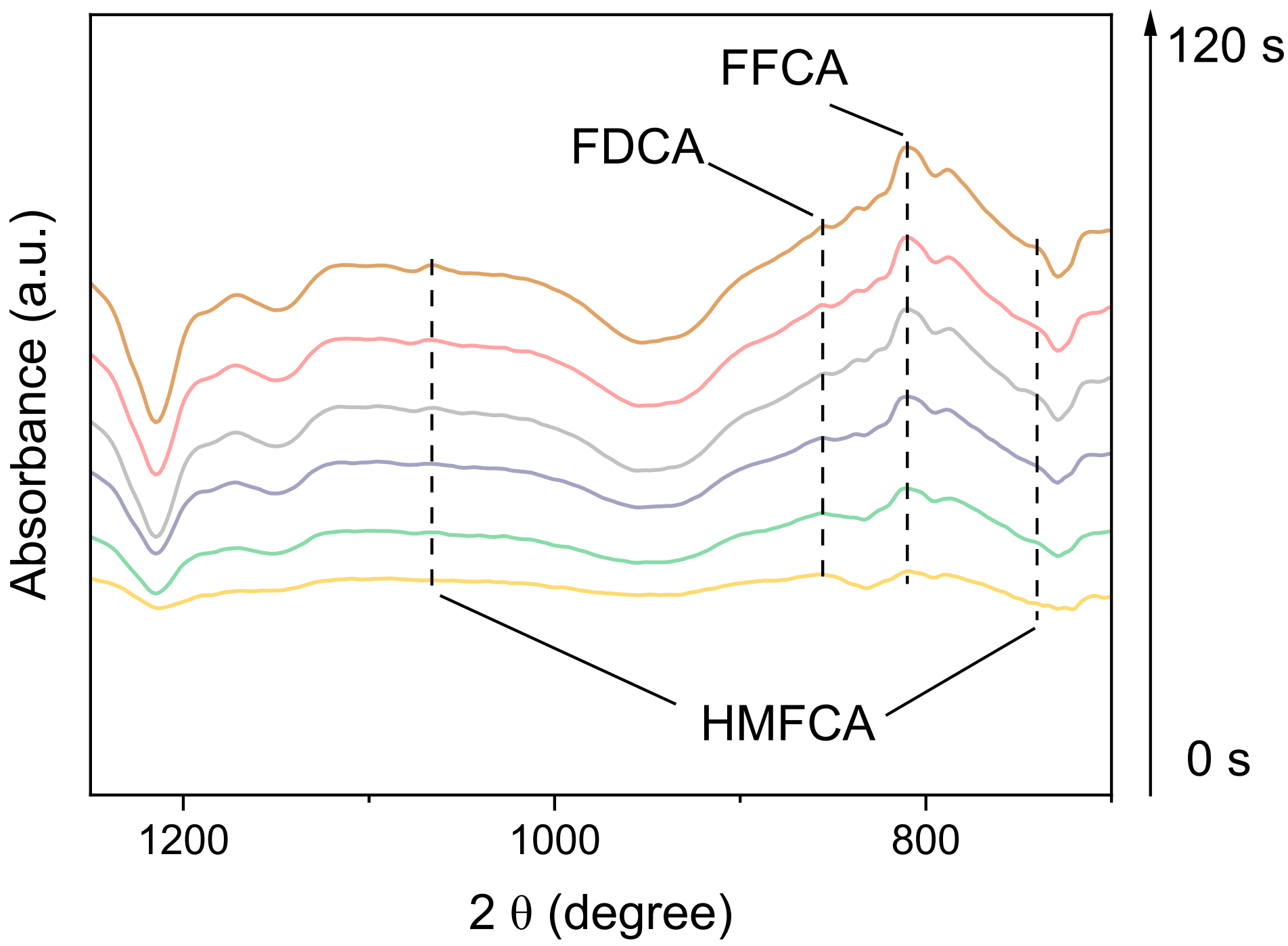

Figure S29 Time-resolved *in situ* ATR-FTIR spectra of $Cu(TFA)_2BPY$ collected at 1.5 V versus RHE during the HMFOR process.

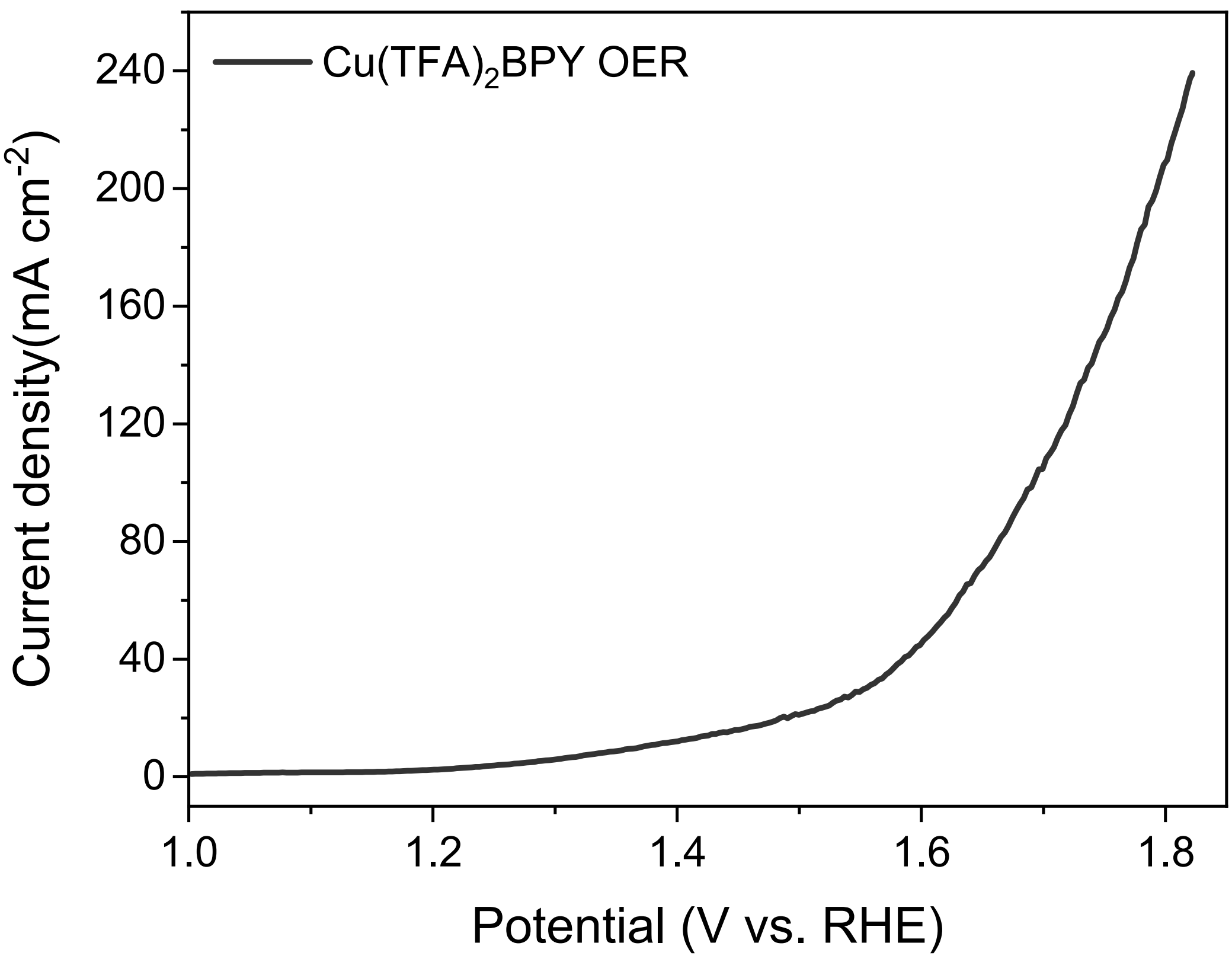

Figure S30 LSV curve of $Cu(TFA)_2BPY$ without HMF in a flow cell.

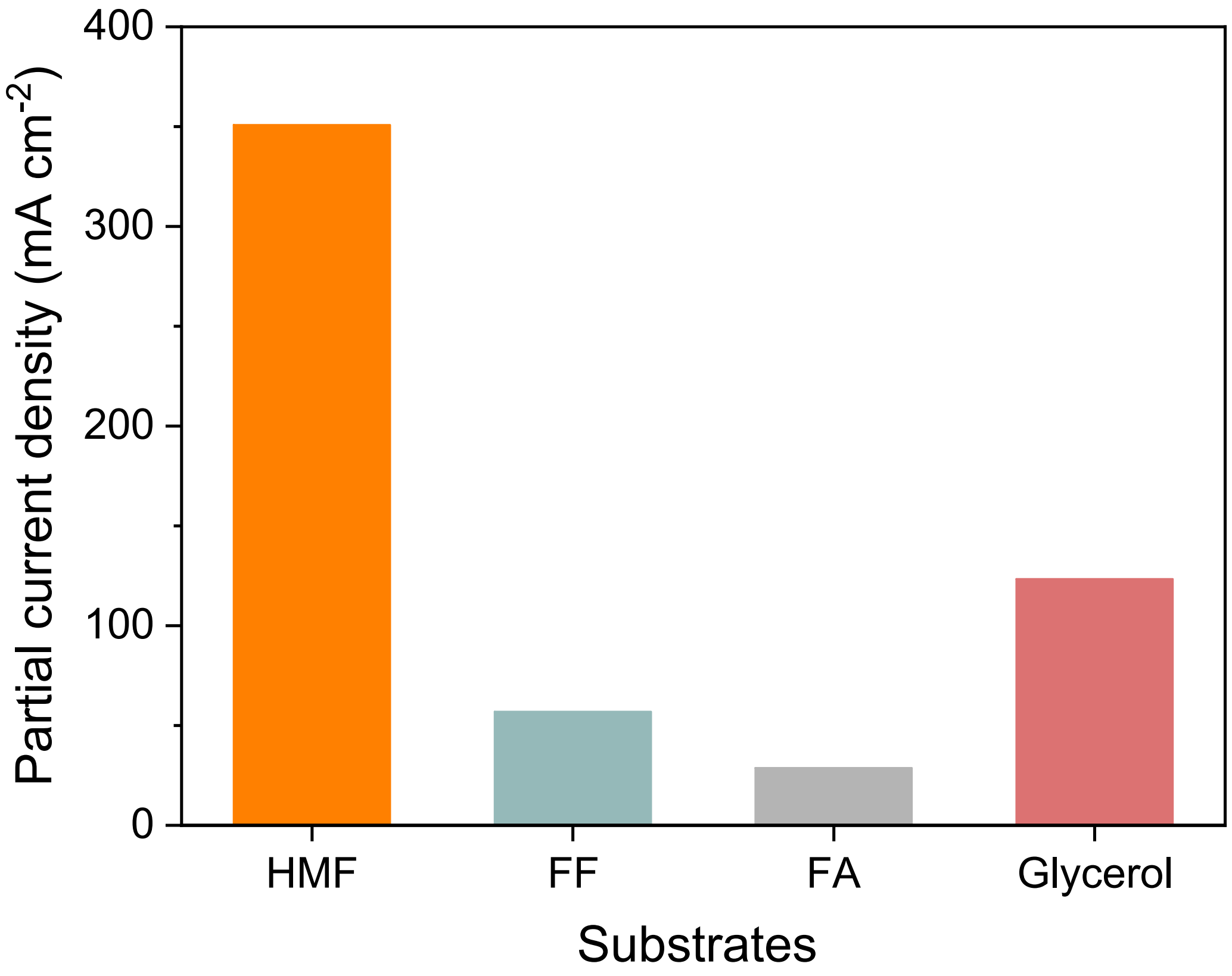

Figure S31 Partial current density of $Cu(TFA)_2BPY$ under HMF, FF, FA and glycerol at 1.6 V vs. RHE.

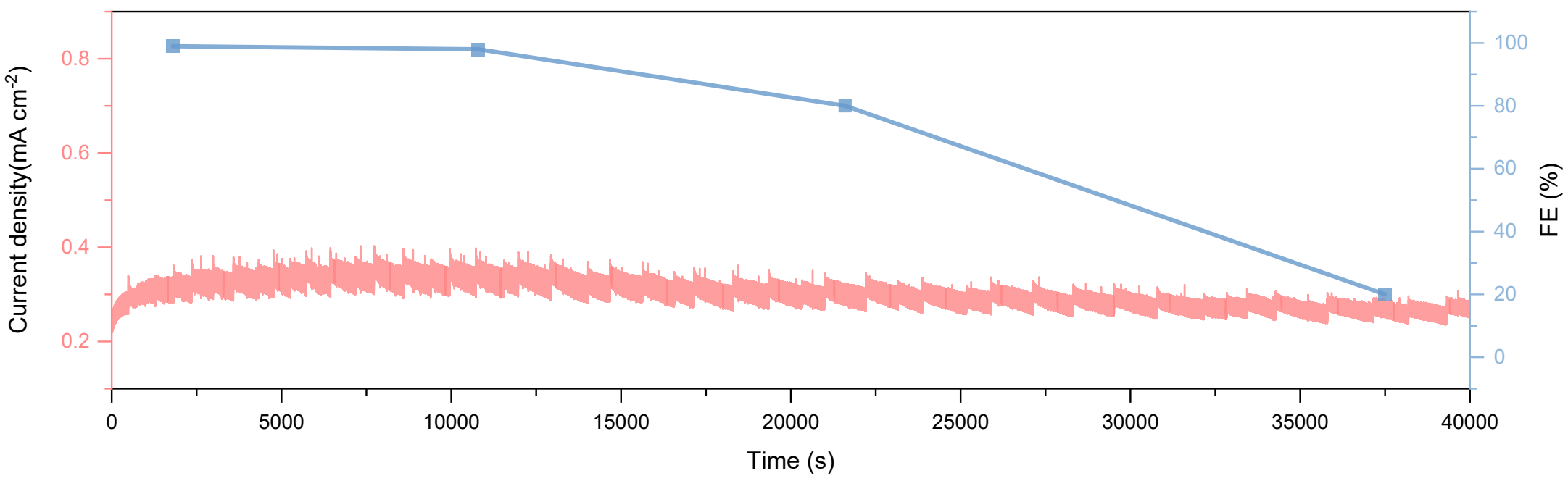

Figure S32 Long-term durability evaluation of $Cu(TFA)_2BPY$ in a flow cell at 1.6 V versus RHE over 40000s. The FE was recorded at 1800s, 10800s, 21600s, and 37500s.

Compounds	$Cu(TFA)_2BPY$
CCDC	2538653
Empirical formula	$C_{14}H_8F_6CuN_2O_4$
Formula weight	445.76
Temperature/K	123(1)
Crystal system	triclinic
Space group	P-1
a/Å	8.4868(8)
b/Å	9.3487(10)
c/Å	10.6659(8)
α/°	112.593(6)
β/°	94.151(6)
γ/°	101.312(4)
Volume/$Å^3$	755.85(13)
Z	1
$\rho_{calc}g/cm^3$	0.979
μ/mm^{-1}	0.771
F(000)	221.0
Crystal size/mm^3	0.14×0.026×0.02
Radiation	MoKα(λ = 0.71073)
Index ranges	$-10 \leq h \leq 11$, $-12 \leq k \leq 12$, $-13 \leq l \leq 13$
Reflections collected	10481
Independent reflections	3304 [R_{int} = 0.1206, Rsigma = 0.1670]
Data/restraints/parameters	3304/0/277
Goodness-of-fit on F^2	0.919
Final R indexes [I>=2σ (I)]	R_1 = 0.0513, wR_2 = 0.0823
Final R indexes [all data]	R_1 = 0.1432, wR_2 = 0.1034
Largest diff. peak/hole/e $Å^{-3}$	0.57/-0.68

Table S1 Crystal data for $Cu(TFA)_2BPY$

Catalyst	Metal	Electrolyte	Potential (V vs RHE)	Concentration of HMF	Main product	FE (%)	Ref.
Ni-HHTP	Ni	1 M KOH	1.5	50 mM	FDCA	~98	[1]
Ni_8PBA	Ni	1 M KOH	-	50 mM	FDCA	99.2	[2]
R-ZIF-CoNi	Co Ni	1 M KOH	1.48	10 mM	FDCA	98.5	[3]
Pd/NiCo-MOF/NF	Pd Ni Co	1 M KOH	1.4	10 mM	FDCA	97.7	[4]
Ni-HITP /PW12-2	Ni W	0.1 M KOH	1.44	10 mM	FDCA	98	[5]
$Ni(TCNQ)_2(H_2O)_2$	Ni	1 M KOH	1.47	50 mM	FDCA	96.9	[6]
$Co(OH)_2$/ Ni-MOF	Co Ni	1 M KOH	1.5	50 mM	FDCA	99.4	[7]
CoNiCu-HAB/NF	Co Ni Cu	1 M KOH	1.4	10 mM	FDCA	95	[8]
CoS/Co-N-C	Co	1 M KOH	1.12	10 mM	FDCA	98.2	[9]
Pt/HEO	Pt	1 M KOH	1.38	10 mM	FDCA	99.8	[10]
NiCoFeMn Al-LDH	Ni Fe Co Mn Al	1 M KOH	1.43	10 mM	FDCA	96.9	[11]
S-NiFe LDH	Ni Fe	1 M KOH	1.36	10 mM	FDCA	96	[12]
$Cu(TFA)_2$-BPY	Cu	0.1 KOH	1.5	10 mM	FDCA	98	This work

Table S2 Comparison of reported molecular catalysts for HMF oxidation in terms of catalytic performance and reaction conditions, along with the catalyst investigated in this work.

Catalysts	Leaching amount wt%
$Cu(OAc)_2BPY$	2.36
$Cu(TFA)_2BPY$	1.25
$Cu(Gly)_2BPY$	3.09

Table S3. ICP-MS analysis of Cu leaching from $Cu(OAc)_2BPY$, $Cu(TFA)_2BPY$, and $Cu(Gly)_2BPY$ after HMFOR.

Assigned species	Wavenumber (cm^{-1})
HMFCA	721
	1010
DFF	796
FFCA	757
FDCA	860

Table S4: Assignment of the characteristic FTIR bands used for tracking the evolution of reactants, intermediates, and products during HMFOR.

Reference

1. Yang, S. W. *et al.* Unraveling the Electrooxidation Mechanism of 5-(Hydroxymethyl)furfural at a Molecular Level via Nickel-Based Two-Dimensional Metal-Organic Frameworks Catalysts. *Acs Catalysis* **14**, 449-462 (2023).
2. Huang, N. Y. *et al.* Rational Design of a Quasi-Metal-Organic Framework by Ligand Engineering for Efficient Biomass Upgrading. *Journal of the American Chemical Society* **147**, 8832-8840 (2025).
3. Shen, Y. *et al.* Promoted Electrochemical Reconstruction of Glassy Metal-Organic Frameworks for Efficient Electrocatalytic 5-Hydroxymethylfurfural Oxidation. *Advanced Energy Materials* **15** (2025).
4. Liu, Z. J. *et al.* Engineering high-valent Ni3+-O active sites through Pd incorporation in NiCo-MOF/Ni-foam for enhanced 5-hydroxymethylfurfural electrooxidation. *Journal of Materials Chemistry A* (2026).
5. Bao, T. *et al.* Highly Ordered Conductive Metal-Organic Frameworks with Chemically Confined Polyoxometalate Clusters: A Dual-Functional Electrocatalyst for Efficient H2O2 Synthesis and Biomass Valorization. *Advanced Materials* **37** (2025).
6. Xiang, T., Chen, K., Ni, S. Q., Wang, X. J. & Chen, C. L. Ligand-engineered Ni-TCNQ framework with synergistic adsorption and charge transfer for efficient HMF oxidation-HER coupling. *Chemical Engineering Journal* **529** (2026).
7. Dai, H. L. *et al.* Highly Self-Healing Co3+/Ni3+ Dual-Active Species for the Electrocatalytic Oxidation of 5-Hydroxymethylfurfural. *Inorganic Chemistry* **63**, 16541-16553 (2024).
8. Zhao, H. Q. *et al.* Boosting biomass upcycling into 2,5-furandicarboxylic acid via amine-induced protonation on ternary metal-organic heterojunction. *Chemical Engineering Journal* **520** (2025).
9. Zhang, J. T. *et al.* Dual Co Sites in n—n Type Heterojunction Enable Selective Electrochemical Co-Valorization of HMF and CO2. *Angewandte Chemie-International Edition* **64** (2025).
10. Ding, G. X. *et al.* Pt-Activated Surface Nucleophilicity on High-Entropy Oxides Enables Superior Biomass Upgrading. *Advanced Functional Materials* **36** (2026).
11. Ding, G. X. *et al.* High-Entropy Regulation of Lattice Oxygen p-Band toward Sustainable Electrocatalytic Biomass Valorization. *Nano Letters* **25**, 8984-8992 (2025).
12. Zhang, J. T. *et al.* Electrooxidation of biomass-derived 5-hydroxymethylfurfural over sulfur-doped nickel-iron layered double hydroxides nanosheets. *Chemical Engineering Journal* **505** (2025).
